# Osteopontin regulates right ventricular failure through integrin ανβ3/PERK/CHOP-dependent inflammatory and apoptotic pathways

**DOI:** 10.3389/fimmu.2025.1569210

**Published:** 2025-05-06

**Authors:** Xiaomei Yang, Xuyang Wang, Kai Li, Qiming Deng, Yonghao Hou, Guangmin Xi, Kangping Lu, Zihua Liu, Yu Bai, Jianbo Wu, Jingui Yu, Peng Zhang

**Affiliations:** ^1^ Department of Anesthesiology, Qilu Hospital of Shandong University, Shandong University, Jinan, Shandong, China; ^2^ National Key Laboratory for Innovation and Transformation of Luobing Theory, Chinese Ministry of Education, Chinese National Health Commission and Chinese Academy of Medical Sciences, Jinan, Shandong, China; ^3^ The Key Laboratory of Cardiovascular Remodeling and Function Research, Chinese Ministry of Education, Chinese National Health Commission and Chinese Academy of Medical Sciences, Jinan, Shandong, China; ^4^ Department of Anesthesiology, Shandong Provincial Qian Foshan Hospital, Shandong University, Jinan, Shandong, China; ^5^ Hypertension Center, Beijing Anzhen Hospital, Capital Medical University, Beijing, China; ^6^ College of Life Science, Qi Lu Normal University, Jinan, Shandong, China; ^7^ The Second Clinical Medical School of Shandong University, Shandong University, Jinan, Shandong, China; ^8^ Department of Hepatobiliary Surgery, The Second Hospital of Shandong University, Shandong University, Jinan, Shandong, China; ^9^ Department of Cardiovascular Surgery, Qilu Hospital of Shandong University, Jinan, Shandong, China; ^10^ Department of Cardiology, Qilu Hospital of Shandong University, Jinan, Shandong, China; ^11^ Department of Anesthesiology, The First Affiliated Hospital of Shandong First Medical University, Jinan, Shandong, China; ^12^ Shandong Institute of Anesthesia and Respiratory Critical Care Medicine, The First Affiliated Hospital of Shandong First Medical University, Jinan, Shandong, China; ^13^ Shandong Provincial Clinical Research Center for Anesthesiology, The First Affiliated Hospital of Shandong First Medical University, Jinan, Shandong, China

**Keywords:** right ventricular failure, osteopontin, endoplasmic reticulum stress, apoptosis, inflammation

## Abstract

**Introduction:**

Right ventricular failure is a life-threatening condition commonly associated with obvious immune responses in its progression. This study aims to investigate the role of osteopontin (OPN) in right ventricular failure pathogenesis and evaluate its potential as a therapeutic target.

**Methods:**

This study adopted a multi-level design. First, immune-related differentially expressed genes (IRDEGs) were identified using the GEO database (GSE161473) and immune cell composition analysis via ImmuCellAI. A right ventricular failure (RVF) rat model was established, and Western blot, RT-qPCR, and immunohistochemical/immunofluorescence analyses were performed to assess OPN expression and inflammatory infiltration. *In vitro*, neonatal rat cardiomyocytes were treated with recombinant OPN to examine changes in endoplasmic reticulum stress markers, while the Integrin-ανβ3 inhibitor LM609 was used to delineate OPN’s mechanism of action. Finally, in a clinical study, serum OPN levels were measured by ELISA and compared with NT-proBNP through correlation and Receiver Operating Characteristic (ROC) analyses.

**Results:**

We found that OPN triggered cardiomyocyte inflammatory responses by activating endoplasmic reticulum stress via the Integrin-ανβ3/PERK/CHOP pathway. OPN exhibited concentration-dependent effects on cardiomyocyte survival: at 2 μg/ml it showed protective effects through BCL-2 modulation, while higher concentrations promoted apoptosis. Importantly, serum OPN levels strongly correlated with NT-proBNP and disease severity in RVF patients.

**Discussion:**

These findings identify OPN as a crucial mediator of RVF pathogenesis through the regulation of inflammatory and apoptotic pathways, establishing its potential as a promising therapeutic target.

## Graphical Abstract

In this study, we investigated the role of OPN in right ventricular failure (RVF) through multiple approaches including bioinformatics analysis, RVF rat model, and clinical patient information. Our findings reveal elevated OPN levels and increased mononuclear macrophage infiltration in RV tissue, accompanied by enhanced endoplasmic reticulum stress signaling that promotes inflammatory responses and cardiomyocyte apoptosis in RVF. Serum OPN levels correlate positively with NT-proBNP, indicating its potential clinical value in RVF.

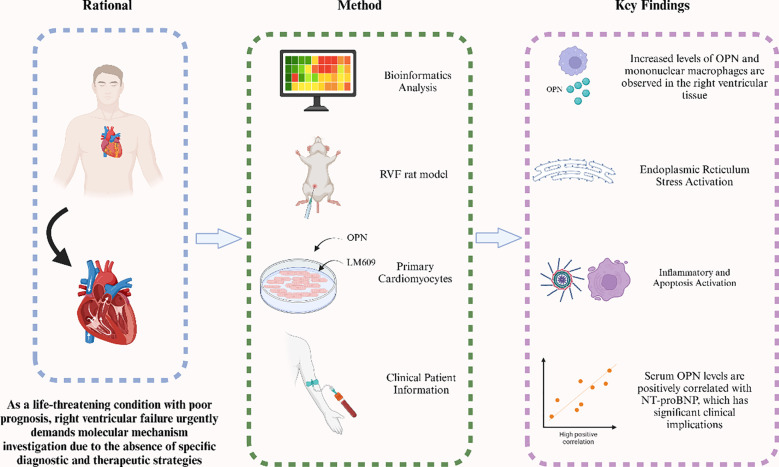

## Highlights

- OPN activates the endoplasmic reticulum stress signaling pathway to promote inflammatory responses and cardiomyocyte apoptosis.
- Serum OPN levels correlate with disease severity and serve as a potential guide for therapeutic strategy optimization.
- This translational study bridges molecular mechanisms with clinical applications, providing new perspectives for RVF diagnosis and treatment.

## Introduction

Right Ventricular Failure (RVF) is a complex syndrome characterized by heart failure signs and symptoms resulting from structural or functional abnormalities of the right ventricle and its associated circulatory system (1–3). Overloaded volume, pressure, or dysfunctional intrinsic myocardial contraction easily leads to RVF (4, 5). Epidemiologically, RVF is relatively prevalent (3), with studies estimating that 3% to 9% of hospitalized acute heart failure (AHF) patients have RVF (6), and the total mortality rate for acute RVF caused by pulmonary arterial hypertension (PAH) can reach 41.3% (7). This highlights the need to delve into the underlying mechanisms of RVF development and progression to formulate more effective therapeutic strategies.

Recent studies have increasingly highlighted the pivotal role of inflammation in RVF pathogenesis. For instance, the NLRP3 inflammasome (NLR Family Pyrin Domain Containing 3) pathway mediated by nuclear factor E2-related factor 2 (Nrf2) is reported in the progression of PAH-induced RVF (8). Additionally, monocyte-derived macrophages in the RV have been shown to activate the NLRP3 inflammasome, further driving inflammatory responses during RVF (9). These findings illuminate excessive inflammatory responses in the development of RVF.

Osteopontin (OPN), also known as secreted phosphoprotein 1 (SPP1), is a secreted phosphorylated glycoprotein found in the extracellular matrix (ECM) and abundantly expressed in tissues such as the kidneys, brain, and bone marrow (10). OPN contributes significantly to physiological processes like cell proliferation, adhesion, migration, immune regulation, and inflammatory responses (11). In recent years, its role in cardiovascular diseases has garnered widespread attention (12). OPN is not expressed in healthy myocardium, it is induced in cardiomyocytes, cardiac fibroblasts, and resident cardiac macrophages under mechanical stresses and hypoxia, accelerating the progression of heart failure (13, 14). Additionally, OPN promotes the recruitment of inflammatory cells, such as macrophages, to the myocardium and contributes to the development and progression of myocardial fibrosis (15). OPN is identified as a key mediator of myocardial hypertrophic response (16) and left ventricular remodeling (17). In vascular remodeling, studies have demonstrated that both genetic ablation of OPN (18) and pharmacological blockade of its receptor integrin-ανβ3 significantly attenuate the neointimal formation of injured arteries (19).

The endoplasmic reticulum (ER) is a multifunctional organelle responsible for protein processing, transport, and signal transduction, playing a vital role in maintaining cellular homeostasis (20). ER stress (ERS) is triggered by the accumulation of unfolded or misfolded proteins within the ER lumen (21), activating the unfolded protein response (UPR) as an adaptive mechanism to restore homeostasis (22). However, chronic activation of UPR can lead to maladaptive responses, impairing cellular function (23, 24). In cardiovascular diseases (CVD), particularly in heart failure, studies have demonstrated the activation of ERS in myocardial cells (25), with structural changes occurring in ER and UPR components due to persistent stimulation (26). Understanding the role of ERS in RVF holds potential therapeutic value, offering new targets and strategies to improve heart failure treatment (27).

In this study, we investigated the role of immune cell infiltration and OPN in RVF, building on our preliminary findings (28). Using bioinformatics analyses, we identified OPN as a key immune-related gene associated with monocyte and macrophage infiltration in RVF. Subsequently, we demonstrated that OPN stimulation activated the endoplasmic reticulum stress signaling pathway, promoting inflammatory response and modulating apoptosis in cardiomyocytes. Furthermore, clinical statistical analysis confirmed a significant correlation between OPN expression levels and disease severity in RVF patients. These findings provide novel insights into the immune mechanisms underlying RVF and highlight OPN as a potential therapeutic target for future clinical interventions.

## Method

The authors declare that all supporting data are available within the article and its **Supplementary Materials**.

### Identification and visualization of differentially expressed immune-related genes from GEO database

The dataset GSE161473 was obtained from the Gene Expression Omnibus (GEO) database and analyzed using the “limma” package in R software. Differentially expressed genes (DEGs) were identified based on specific criteria: |log2 fold change (FC)| > 2 and *P-value* < 0.05. All immune-related genes were retrieved from the Immunology Database and Analysis Portal (ImmPort, https://www.immport.org/home). Immune-related differentially expressed genes (IRDEGs) were identified by matching the immune-related genes from the ImmPort database with the DEGs. The ‘ggplot2’ package in R was used to create a volcano plot of the DEGs, and the ‘heatmap’ package was employed to construct heatmaps for both IRDEGs and DEGs.

### Cross-validation analysis of IRDEGs

We obtained the GSE120852 dataset (from the GEO database) and gene sequencing data from the right ventricular tissues of RVF rat models to validate the reliability of immune-related differentially expressed genes. The GSE120852 dataset contained 5 RVF samples and 5 control samples. Similarly, the rat transcriptome sequencing samples included 5 RVF model groups and 5 sham-operated control groups. Using the same methodology described above, we screened for immune-related genes in both datasets and performed statistical significance analysis. Through cross-validation comparison, we identified target genes that showed consistent differential expressions.

### Gene ontology analysis of differentially expressed genes in RVF

To explore the biological functions of the immune-related differentially expressed genes (IRDEGs) and differentially expressed genes (DEGs), Gene Ontology (GO) enrichment analysis was conducted using the R package “Cluster Profiler” (version 3.10.1) (29). The immune genes from the signature were used as the gene list with the entire transcriptome as the background. GO terms were categorized into biological process (BP), cellular component (CC), and molecular function (MF). Functions with an adjusted *p-value* < 0.05 were considered significantly enriched GO categories.

### Evaluation of immune cell infiltration and correlation analysis

Using the CIBERSORT tool in R, we filtered samples with *P* < 0.05 and obtained an immune cell infiltration matrix containing 22 types of immune cells, along with their proportions. The “ggplot2” package was employed to visualize this immune cell infiltration matrix. The correlation between key immune genes and immune cells was analyzed using the ‘Corrplot’ package in R. To explore potential relationships between key immune-related genes (IRGs) and infiltrating immune cells, Spearman correlation analysis was conducted and visualized using the “ggpubr” package.

### Experimental animals

*Male Sprague-Dawley rats* (6–8 weeks old, 220~250g) were obtained from the Center of Experimental Animals at Shandong University (Jinan, China) (Number ECAESDUSM 2012029). Animals were maintained under standard conditions (20 ± 2°C, 55 ± 5% humidity, 12-hour light/dark cycle) with free access to food and water. All experimental procedures were approved by the Institutional Animal Care and Use Committee of Shandong University and conducted following the 3R principles. The PAH-induced RVF model was established by a single intraperitoneal injection of monocrotaline (60 mg/kg), while control rats received saline injection (9, 30). All rats were monitored for 4–5 weeks after injection. For the RVF model, Sprague-Dawley rats were randomly allocated into Control and MCT-induced groups (n = 5 per group). Primary cardiomyocytes were isolated from 30 neonatal rats (3-day-old) across three independent experiments.

### Immunohistochemistry and immunofluorescence staining of right ventricle

Immunohistochemistry and immunofluorescence staining were performed on 5-μm paraffin sections of right heart tissue. After deparaffinization and rehydration through graded ethanol, antigen retrieval was performed. Endogenous peroxidase activity was blocked with 3% H_2_O_2_ for 15 minutes at room temperature. Sections were blocked with normal goat serum for 30 minutes and then incubated with primary antibodies against OPN (1:200, sc-21742, Santa Cruz) and CD68 (1:200, 97778, Cell Signaling Technology) overnight at 4°C. After PBS washing, sections were incubated with secondary antibody for 45 minutes at room temperature. The immunoreactivity was visualized using DAB chromogen and counterstained with hematoxylin. For IF staining, after PBS washing, sections were incubated with fluorescent-labeled secondary antibodies for 1 hour at room temperature in the dark. Nuclei were counterstained and mounted using a DAPI-containing mounting medium (ab104139, Abcam). Images were captured using a confocal microscope and analyzed with Image J software.

### TUNEL staining

Rats’ right ventricular myocardial tissues were fixed in 4% paraformaldehyde for 24 hours at room temperature, dehydrated through a graded ethanol series, cleared in xylene, and embedded in paraffin. Coronal tissue sections (5 μm thickness) were cut using a microtome and mounted onto glass slides. After deparaffinization in xylene and rehydration through graded ethanol series (100%, 95%, 85%, and 75%), sections were treated with proteinase K (20 μg/mL) for 15 minutes at room temperature for antigen retrieval. According to the manufacturer’s instructions, apoptosis cells were detected using a TUNEL detection kit (Roche). Briefly, sections were incubated with a TUNEL reaction mixture containing terminal deoxynucleotidyl transferase and fluorescein-dUTP for 60 minutes at 37°C in a humidified chamber in the dark. Nuclei were counterstained with DAPI (1:1000) for 5 minutes. Images were captured using a fluorescence microscope (400×) with appropriate filters for red (TUNEL-positive cells) and blue (DAPI) channels. Five random fields per sample were photographed, and the percentage of TUNEL-positive cells was calculated as the number of TUNEL-positive cells divided by the total number of DAPI-positive cells.

### Isolation and cultivation of primary neonatal rat cardiomyocytes

Primary neonatal rat cardiomyocytes (NRVMs) were isolated following the protocol described by Lange et al. (31). Briefly, hearts from 3-day-old neonatal rats (n=10) were harvested and minced in pre-cooled D-Hanks buffer (H1045, Solarbio). The tissue fragments were enzymatically digested with 0.075% collagenase II solution (LS004176, Worthington Biochemical) at 37°C with gentle stirring (100–200 rpm) for approximately 1 hour. After sequential digestion and filtration through a 100-μm cell strainer, cardiomyocytes were separated from cardiac fibroblasts using the pre-plating technique. The non-adherent cardiomyocytes were collected after 1.5 hours and treated with 5-BrdU (0.1 mmol/L) to inhibit residual fibroblast proliferation. Cells were seeded in gelatin-coated plates (2×10^4^ cells/well for 96-well plates or 2-3×10^6^ cells/well for 6-well plates) and maintained in DMEM supplemented with 10% fetal calf serum, 100 μg/ml streptomycin, and 100 U/ml penicillin at 37°C in a humidified atmosphere with 5% CO_2_ for 48 hours. The cardiomyocytes were then treated with OPN (HY-P70499, MCE) at various concentrations (1, 2, and 5 μg/ml) for 24 hours for subsequent experiments.

### CCK-8 assay

Cell viability was assessed using CCK8 assay (GK10001, GLPBIO). Cardiomyocytes were treated with OPN (HY-P70499, MCE) at different concentrations (1, 2, and 5 μg/ml) for 24 hours. CCK-8 solution (10 μl/well) was added 2 hours before the end of treatment, and the absorbance at 450 nm was measured using a microplate reader (1681130, Bio-Rad).

### Total RNA extraction and RT-PCR analysis

Total RNA was extracted from rat right ventricular tissue (4–5 mg) and cultured primary cardiomyocytes using an RNA fast200 kit (220011, Fastagen) according to the manufacturer’s protocol. RNA concentration was measured using a Nanodrop spectrophotometer (Thermo Fisher Scientific). First-strand cDNA was synthesized from 1 μg total RNA using HiScript III RT SuperMix (R323-01, Vazyme Biotech). Quantitative PCR was performed using ChamQ SYBR qPCR Master Mix (Q311-02, Vazyme Biotech) on CFX Connect Real-Time PCR System (Bio-Rad). Gene expression was normalized to GAPDH. Primer sequences are listed in **Supplementary Table 2**.

### Western blot analysis

Total protein was extracted from rat right ventricular myocardial tissue and cultured primary myocardial cells using enhanced RIPA lysis buffer (P0013B, Beyotime) supplemented with PMSF (ST506, Beyotime) and phosphatase inhibitor (04906837001, Roche). Protein concentration was determined using the BCA Protein Quantification Kit (CW0014S, Cwbiotech). Equal amounts of protein (30μg) were separated by 10%-12% SDS-PAGE and transferred to PVDF membranes (1620177, Bio-Rad). After blocking with 5% non-fat milk for 1–2 h at room temperature, membranes were incubated overnight at 4°C with primary antibodies against OPN (1:200, sc-21742, Santa Cruz), PERK (1:1000, 3192S, CST), CHOP (1:1000, 2895T, CST), TXNIP (1:2000, 14715S, CST), NLRP3 (1:2000, ab263899, Abcam), GSDMD (1:3000, 20770-1-AP, Proteintech), Caspase-1 (1:3000, 22915-1-AP, Proteintech), IL-1β (1:3000, 16806-1-AP, Proteintech), BAX (1:3000, 50599-2-Ig, Proteintech), BCL-2 (1:2000, 26593-1-AP, Proteintech), Caspase-3 (1:1000, 19677-1-AP, Proteintech), and GAPDH (1:10000, 10494-1-AP, Proteintech). After washing with TBST, membranes were incubated with HRP-conjugated secondary antibodies (anti-rabbit: 7074S, CST, 1:2000; anti-mouse: ZB-2305, ZSGB-Bio, 1:10000) for 1 h at room temperature. Protein bands were visualized using SuperPico ECL Kit (E422, Vazyme) and captured by a chemiluminescence imaging system (Tanon 5200). Band intensities were quantified using Image J software and normalized to GAPDH. Experimental group ratios were normalized to the control group (set as 1.0) to account for inter-blot variability in GAPDH levels or protein loading. Statistical analyses were based on these normalized values.

### Patients

This study was conducted at Qilu Hospital of Shandong University and approved by the institutional Ethics Committee (2021-299). Ten patients with confirmed PAH-RVF and ten age- and gender-matched healthy controls were enrolled between May 2023 and May 2024. Inclusion criteria for PAH-RVF patients required: (1) Diagnosis of PAH confirmed by right heart catheterization (mPAP ≥25 mmHg at rest); (2) RVF defined by echocardiographic evidence of RV dilation (RV basal diameter >42 mm) and reduced systolic function (TAPSE <17 mm); (3) Presence of ≥1 clinical sign/symptom of right heart failure (e.g., peripheral edema, jugular venous distension, or hepatomegaly). Exclusion criteria: (1) severe renal dysfunction (estimated glomerular filtration rate, eGFR <60 mL/min/1.73 m²); (2) chronic liver disease (Child-Pugh class B/C or ALT/AST >3× upper limit of normal); (3) active infection (C-reactive protein >10 mg/L), autoimmune diseases, or malignancy; (4) History of left ventricular dysfunction (LVEF <50% or echocardiographic evidence of diastolic dysfunction); (5) Significant left-sided heart disease (e.g., prior myocardial infarction, cardiomyopathy, or moderate-to-severe valvular disease). Written informed consent was obtained from all participants. Blood samples were collected via antecubital venipuncture after overnight fasting. The serum was separated by centrifugation (3000 rpm, 15 min) and stored at -80°C until analysis. Serum NT-proBNP levels were measured by chemiluminescent immunoassay. Serum OPN levels were measured using the Human OPN ELISA Kit (EK0482, BOSTER) according to the manufacturer’s instructions. Briefly, 100 μl of serum samples or pre-diluted standards were added to antibody-coated plates, followed by sequential incubation with biotin-labeled antibody working solution, ABC working solution, and TMB substrate. After adding the stop solution, optical density was measured at 450 nm with 630 nm as the reference wavelength. All samples were tested in duplicate, and serum concentrations were determined using standard curves constructed from gradient-diluted standards. Echocardiographic parameters were assessed using Vivid T9 (GE Healthcare, USA). Key measurements included Tricuspid Annular Plane Systolic Excursion (TAPSE) and Pulmonary Artery Systolic Pressure (PASP), with the latter calculated from peak tricuspid regurgitation gradient plus estimated right atrial pressure.

### Statistical analysis

Data was analyzed using GraphPad Prism 9.0. Continuous variables were presented as mean ± standard deviation (SD) for normally distributed data or median [interquartile range, IQR] for non-normally distributed data. The two groups were compared using the student’s t-test for normally distributed data or the Mann-Whitney U test for non-normally distributed data. In contrast, multiple group comparisons were analyzed by one-way ANOVA or the Kruskal-Wallis test as appropriate. Correlations were assessed using Pearson’s correlation coefficient. Receiver Operating Characteristic (ROC) curves and area under the curve (AUC) were calculated to evaluate diagnostic performance. *P*<0.05 was considered statistically significant.

## Result

### Screening of differentially expressed genes and immune-related genes in right ventricular tissues of RVF patients

**Figure 1:**
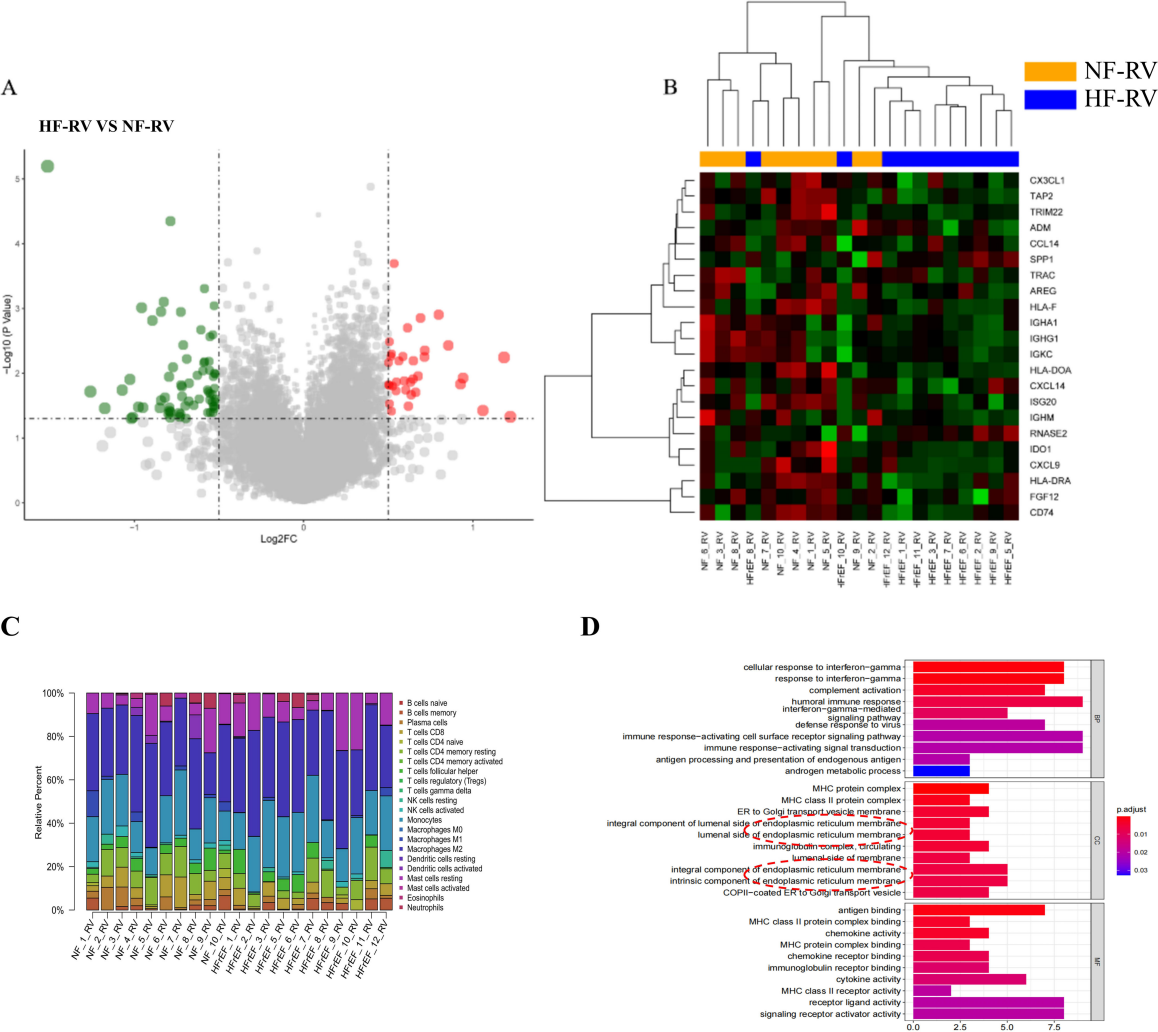
Bioinformatic analysis of RVF patient samples (GSE161473). **(A)** Volcano plot of 109 differentially expressed genes (DEGs) between HF-RV (n=11) and NF-RV (n=10) groups from the GSE161473 database, analyzed using R programming. Red dots represent significantly upregulated genes, while green dots indicate significantly downregulated genes (cut-off criteria: |log2 fold change (FC)| > 2, *P* < 0.05). **(B)** Heatmap visualization of 22 immune-related differentially expressed genes (IRDEGs) identified by intersecting DEGs with immune-related gene sets. Groups NF-RV and HFrEF-RV are labeled as NF-RV and HF-RV, respectively. **(C)** Immune cell composition in right ventricular tissues from normal (NF-RV) and heart failure (HF-RV) patients. Colors denote distinct immune cell populations; the y-axis shows relative percentages (summing to 100% per sample). **(D)** GO enrichment analysis of differentially expressed genes. Color intensity corresponds to adjusted *P*-values. Analysis dimensions include Biological Process (BP), Cellular Component (CC), and Molecular Function (MF). CX3CL1, Chemokine C-X3-C Motif Ligand 1; TAP2, Transporter Associated with Antigen Processing 2; TRIM22, Tripartite Motif Containing 22; ADM, (Adrenomedullin; CCL14, C-C Motif Chemokine Ligand 14; SPP1/OPN, Secreted Phosphoprotein 1/Osteopontin; TRAC, T Cell Receptor Alpha Constant; AREG, (Amphiregulin; HLA-F, Major Histocompatibility Complex, Class I, F; IGHA1, Immunoglobulin Heavy Constant Alpha 1; IGHG1, Immunoglobulin Heavy Constant Gamma 1; IGKC, Immunoglobulin Kappa Constant; HLA-DOA, Major Histocompatibility Complex, Class II, DO Alpha; CXCL14, C-X-C Motif Chemokine Ligand 14; ISG20, Interferon Stimulated Exonuclease Gene 20; IGHM, Immunoglobulin Heavy Constant Mu; RNASE2, Ribonuclease A Family Member 2; IDO1, Indoleamine 2,3-Dioxygenase 1; CXCL9, C-X-C Motif Chemokine Ligand 9; HLA-DRA, Major Histocompatibility Complex, Class II, DR Alpha; FGF12, Fibroblast Growth Factor 12; CD74, CD74 Molecule.

The bioinformatics analysis workflow is illustrated in **Supplementary Figure 1**. We initially analyzed transcriptomic datasets from RVF patients’ and normal controls’ right ventricular tissues. Using stringent screening criteria (|log2 fold change (FC)| > 2 and *P* < 0.05), we identified 109 differentially expressed genes (DEGs) (**Figure 1A**). Subsequently, through intersection analysis with the immune-related gene database, 22 immune-related differentially expressed genes (IRDEGs) were identified (**Figure 1B**). Detailed information about IRDEGs is provided in **Supplementary Table 1**. Using the ImmuCellAI algorithm (32), we estimated the relative proportions of immune cell types between the control and RVF groups (**Figure 1C**). Furthermore, we performed Gene Ontology (GO) enrichment analysis on differentially expressed genes (DEGs) (**Figure 1D**), which provided valuable insights for subsequent experimental investigations.

### Identification and validation of OPN

**Figure 2:**
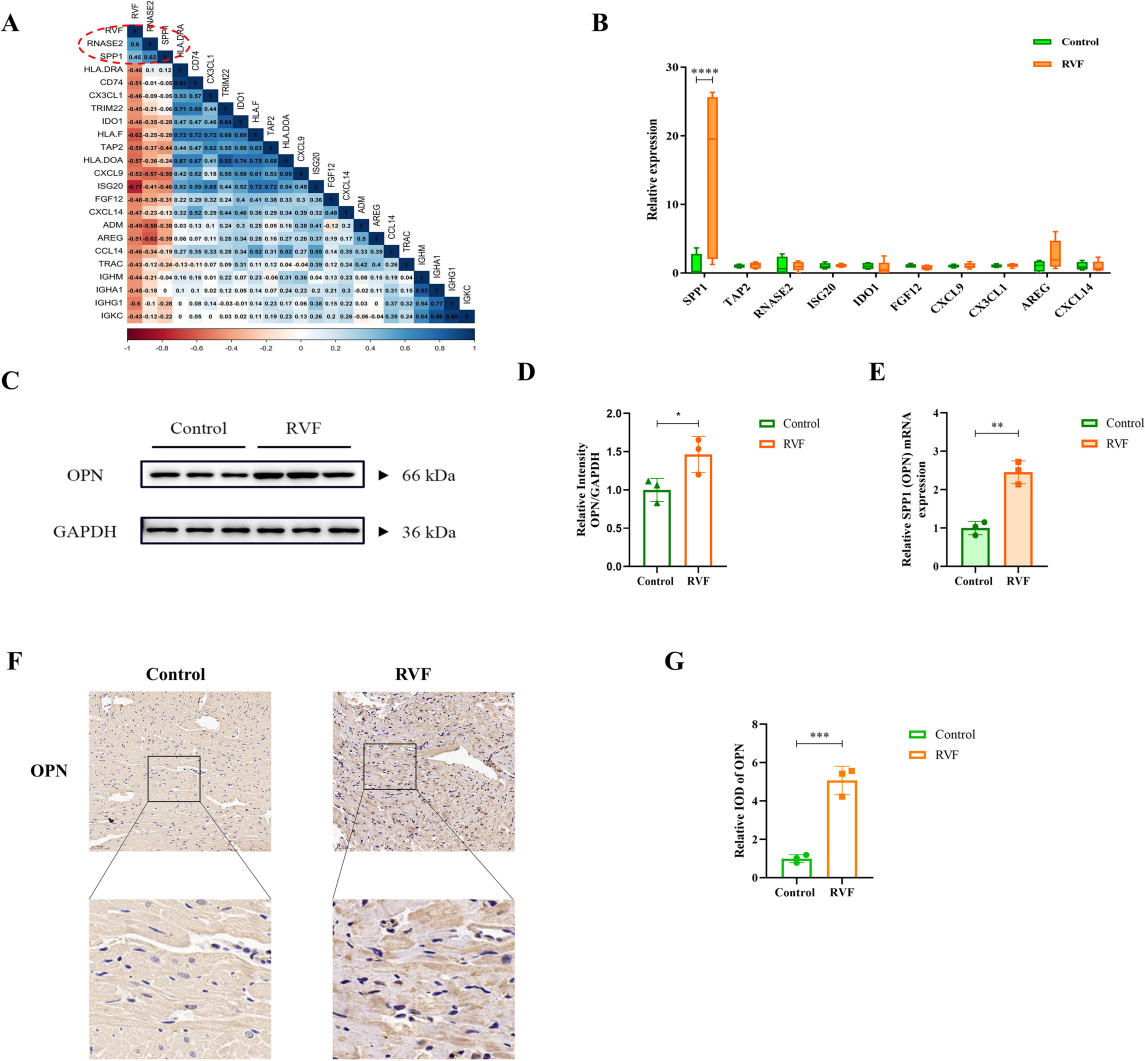
OPN expression analysis in RVF right ventricular tissue. **(A)** Pearson correlation matrix between RVF severity and immune-related genes. The color scale ranges from dark red (correlation coefficient 1) to dark blue (correlation coefficient 1). Numbers indicate correlation coefficients for selected genes (e.g., RNASE2 and SPP1). **(B)** Box plots of IRDEGs expression from RVF rat transcriptome data. Green and orange boxes represent the Control and RVF groups, respectively. Vertical bars denote data ranges(n=4-5). **(C, D)** Western blot analysis of OPN protein in rat right ventricular tissue (n=3). Densitometric quantification was performed using ImageJ software. **(E)** RT-qPCR analysis of SPP1 (OPN) mRNA levels in Control and RVF groups (n=3 rats/group). **(F, G)** Representative immunohistochemical staining of OPN in Control and RVF groups (upper panels: full view; lower panels: magnified regions; n=3 rats/group). Scale bars: 50 μm (×200 magnification). Data are presented as mean ± SD. Statistical significance is denoted by asterisks (^*^ *P* < 0.05, ^**^ *P* < 0.01, ^***^ *P* < 0.001, ^****^ *P* < 0.0001).

Correlation analysis between IRDEGs and the RVF database (GSE161473) (**Figure 2A**) revealed that SPP1 (OPN) and RNASE2 showed positive correlations with RVF, with Pearson correlation coefficients of 0.45 and 0.60, respectively. To validate these findings, we identified 17 and 10 overlapping IRDEGs in the GSE120852 database and RVF rat right ventricle sequencing dataset, respectively (**Supplementary Figure 2**, **Figure 2B**). Our sequencing data analysis from RV of rats (**Figure 2B**) demonstrated significant upregulation of SPP1 (*P* < 0.0001). These findings led us to focus our subsequent investigations on the SPP1 gene and its encoded protein OPN.

### Significant upregulation of OPN expression in RVF right ventricular tissues

To further investigate the findings, we established an RVF rat model and collected the right ventricular tissues. Western blot and RT-PCR analyses revealed significantly elevated levels of both OPN (*P* = 0.0485) and SPP1 (OPN) mRNA expression (*P* = 0.0019) in the right ventricular of RVF rats compared to controls (**Figures 2D–F**). Immunohistochemical staining further confirmed the upregulation of OPN expression in RVF right ventricular tissues.

### Enhanced monocyte-macrophage infiltration in RVF right ventricle

**Figure 3:**
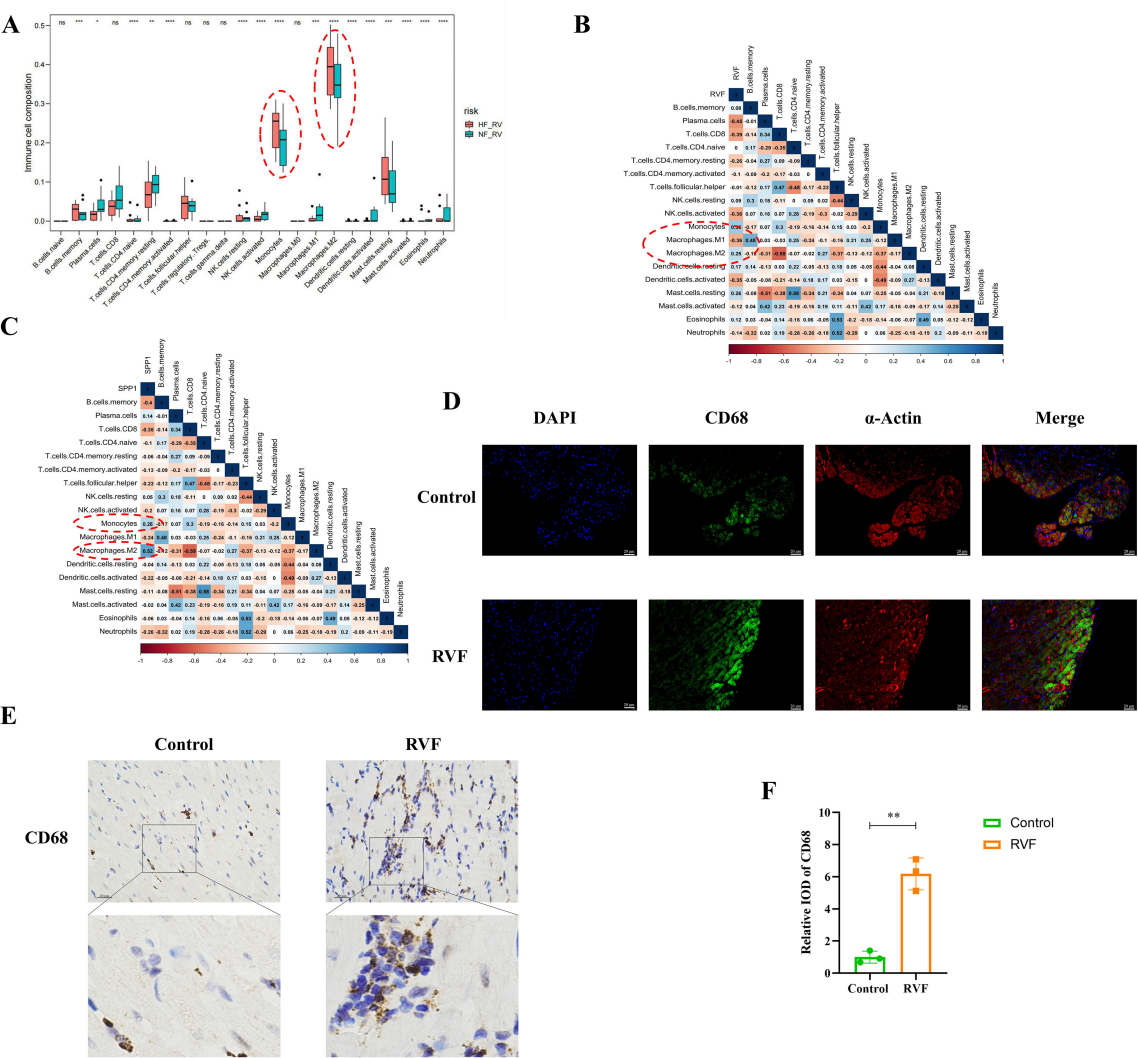
Immune cell infiltration and correlation analysis in RVF. **(A)** Box plots showing relative proportions of immune cell populations in NF-RV (green) and HF-RV (red) groups, estimated using the ImmuCellAI algorithm. Vertical bars indicate data ranges. **(B, C)** Correlation analysis between immune cell subsets and SPP1 (OPN) expression **(B)** or RVF severity **(C)**. Color gradients range from dark red (correlation coefficient 1) to dark blue (correlation coefficient 1). **(D)** Immunofluorescence co-staining of CD68 (green) and α-Actin (red) in Control and RVF groups. Nuclei were counterstained with DAPI (blue). Scale bar: 20 μm (×400 magnification). **(E, F)** Representative immunohistochemical staining of CD68 in rat right ventricular tissue (upper panels: whole-field images; lower panels: magnified views of boxed areas); The bar graph illustrates the quantification of CD68 expression. Scale bars: 20 μm (×400 magnification). n = 3 rats/group. Data are presented as mean ± SD. Statistical significance is denoted by asterisks *P < 0.05, **P < 0.01, ***P < 0.001, ****P < 0.0001, ns: not significant.

Regarding immune cell composition, we employed the ImmuCellAI algorithm to estimate the relative proportions of immune cell types in control and heart failure groups within our dataset (**Figure 1C**). Analysis revealed significant differences in 20 immune cell types between groups, with notably higher levels of monocytes, M2 macrophages, memory B cells, resting NK cells, and resting mast cells in the RVF group compared to controls (**Figure 3A**). Subsequent correlation analyses (**Figures 3B, C**) of SPP1 (OPN) with immune cells demonstrated positive correlations with monocytes (R = 0.28) and M2 macrophages (R = 0.52). Similar correlation patterns were observed between these immune cells and the RVF (R = 0.36 and R = 0.25, respectively). Further validation through immunohistochemistry and immunofluorescence staining (**Figures 3D–F**, **Supplementary Figure 3**) confirmed significantly increased monocyte-macrophage infiltration in the right ventricular tissues of the RVF group compared to controls. These findings collectively suggest a potentially crucial role of monocyte-macrophages in the progression of RVF.

### OPN modulates cardiomyocyte apoptosis and induces endoplasmic reticulum stress

**Figure 4:**
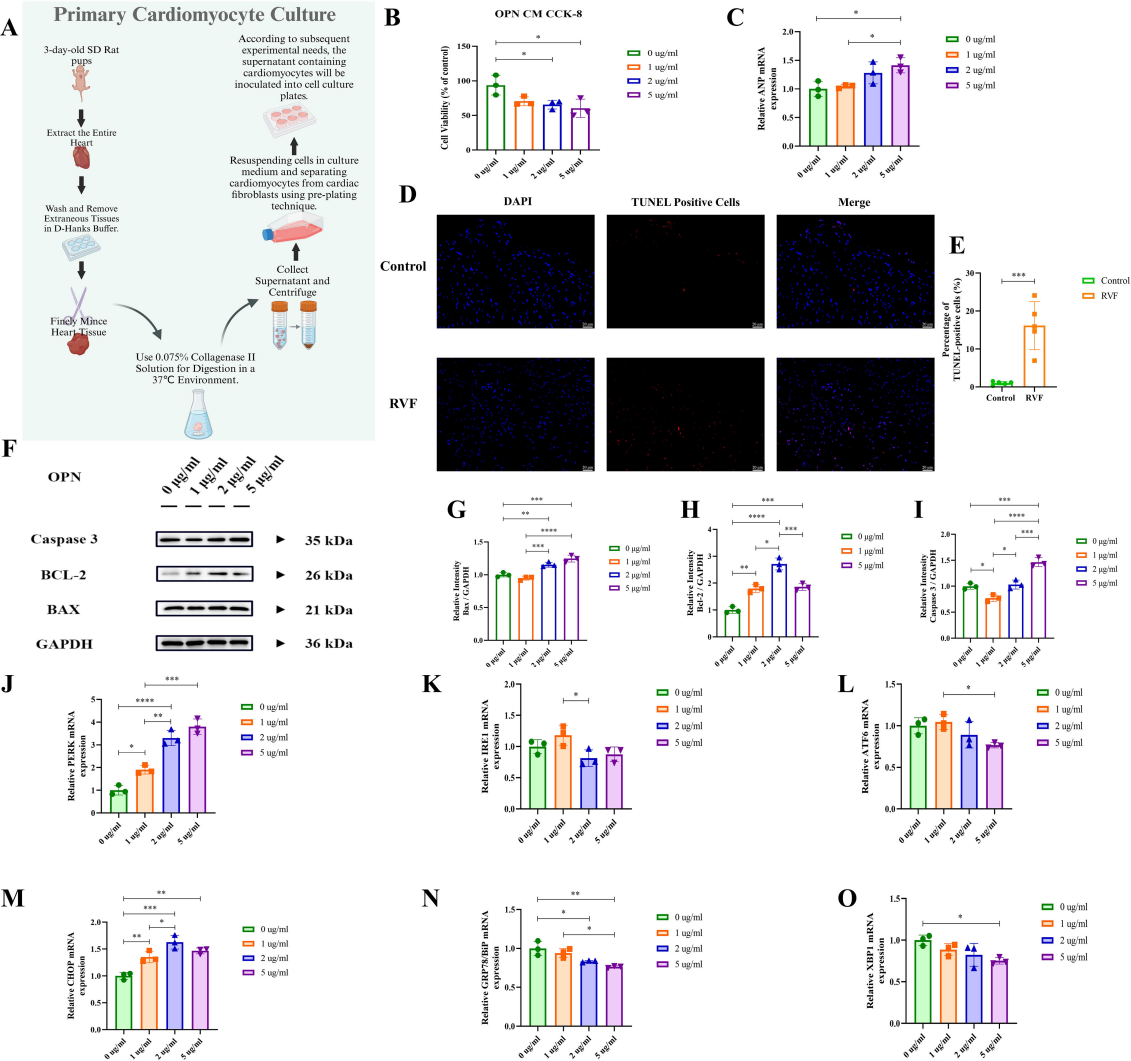
Effects of OPN on neonatal primary cardiomyocytes. **(A)** Schematic workflow for isolation and culture of neonatal rat primary cardiomyocytes. **(B)** Cell viability was assessed by CCK-8 assay under treatment with OPN (0, 1, 2, 5 μg/ml) for 24h. **(C)** RT-qPCR analysis of *ANP* mRNA expression in cardiomyocytes treated with OPN (0, 1, 2, 5 μg/ml). **(D, E)** TUNEL staining of apoptotic cells (red) in rat right ventricular tissue (Control vs. RVF). Nuclei counterstained with DAPI (blue). Scale bar: 20 μm (×400 magnification). n = 5 rats/group. **(F–I)** Western blot analysis of apoptosis-related proteins (BAX, BCL-2, Caspase-3) in cardiomyocytes treated with OPN (0, 1, 2, 5 μg/ml). Protein levels normalized to GAPDH. **(J–O)** RT-qPCR analysis of endoplasmic reticulum stress (ERS) markers (*PERK*, *CHOP*, *IRE1*, *ATF6*, *GRP78*, *XBP1*) in cardiomyocytes treated with OPN (0, 1, 2, 5 μg/ml). PERK, Protein kinase R (PKR)-like Endoplasmic Reticulum Kinase; IRE1, Inositol-Requiring Enzyme 1; ATF6, Activating Transcription Factor 6; CHOP, C/EBP Homologous Protein; GRP78, Glucose-Regulated Protein 78; XBP1, X-Box Binding Protein 1; BAX, BCL2 Associated X Protein; Caspase 3, Cysteine-ASPartic acid proteASE 3; BCL-2, B-Cell Lymphoma 2. Data are presented as mean ± SD. Statistical significance: ^*^ *P* < 0.05, ^**^ *P* < 0.01, ^***^ *P* < 0.001, ^****^ *P* < 0.0001.

To examine the effects of OPN on cardiomyocytes, we designed experiments based on the protocols of Rotem et al. (33) and Meng et al. (34), treating cells with varying OPN concentrations (**Figure 4A**). CCK-8 cell viability assay (**Figure 4B**), and ANP mRNA expression analysis (**Figure 4C**) demonstrated that increasing OPN concentrations induced cellular stress and injury, reduced cell viability, and promoted pathological remodeling. Notably, at 5 μg/ml OPN, cell viability decreased by approximately 50% (*P* = 0.0214). TUNEL staining of right ventricular tissue (**Figures 4D, E**) and Western blot analysis of apoptosis-related proteins (**Figures 4F–I**) indicated that OPN regulates cell survival primarily through BCL-2 expression modulation, with 2 μg/ml emerging as a threshold concentration where cells exhibited significant protective effects. Exceeding the concentration of 2 μg/ml, OPN induces cardiomyocyte apoptosis. To clarify the underlying mechanisms, we performed GO functional enrichment analysis of differentially expressed genes (DEGs) (**Figure 1C**). The results revealed significant enrichment in endoplasmic reticulum-related immune responses, suggesting the activation of endoplasmic reticulum stress (ERS) in RVF. Subsequently, we examined six key ERS molecules (PERK, CHOP, IRE1, ATF6, GRP78, and XBP1) using RT-PCR (**Figures 4J–O**). Our analysis demonstrated that OPN upregulates PERK and CHOP expression (**Figures 4J, M**). These results, combined with previous findings (35), confirmed that OPN mediates the ERS response through the PERK-CHOP pathway. Our findings demonstrated the dual regulatory role of OPN in ERS and the apoptosis of cardiomyocytes.

### OPN induces cardiomyocyte inflammation via ERS pathway, partially reversed by integrin-ανβ3 inhibitor

**Figure 5:**
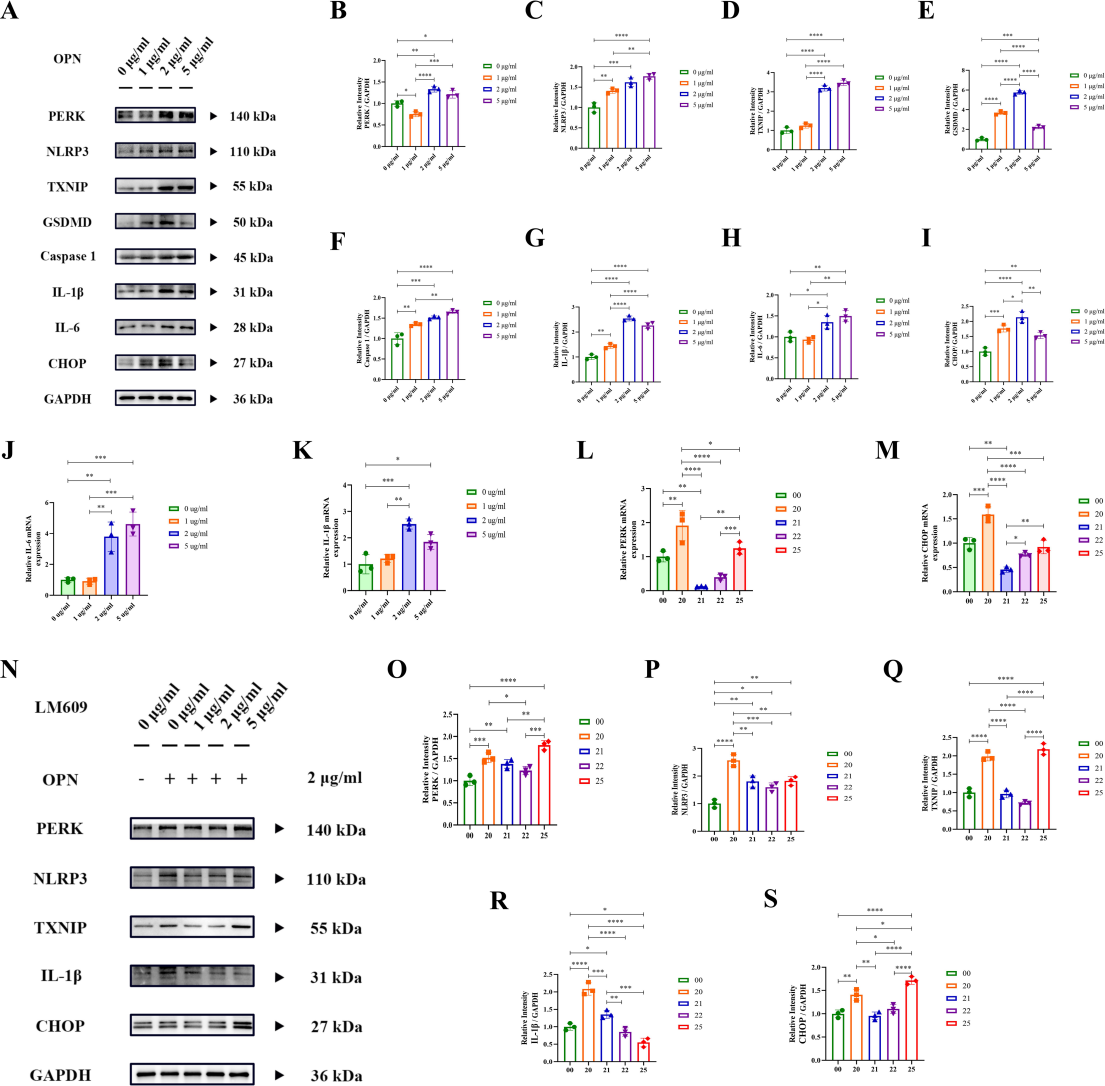
OPN activates PERK-NLRP3 inflammatory pathway via integrin-ανβ3. **(A–I)** Western blot analysis of ER stress (PERK, CHOP) and inflammatory pathway proteins (NLRP3, TXNIP, GSDMD, Caspase-1, IL-1β, IL-6) in cardiomyocytes treated with OPN (0, 1, 2, 5 μg/ml). Protein levels normalized to GAPDH. **(J, K)** RT-qPCR analysis of *IL-1β* and *IL-6* mRNA expression in cardiomyocytes treated with OPN (0, 1, 2, 5 μg/ml) for 24h. **(L, M)** RT-qPCR analysis of ER stress genes (*PERK, CHOP*) in cardiomyocytes treated with LM609 (integrin-ανβ3 neutralizing antibody) at indicated concentrations (0, 1, 2, 5 μg/ml). **(N–S)** Western blot analysis of ER stress and inflammatory proteins in cardiomyocytes treated with LM609 (0, 1, 2, 5 μg/ml) and OPN (2 μg/ml). Protein levels normalized to GAPDH. Experimental groups: Group 00: 0 μg/ml OPN + 0 μg/ml LM609, group 20 (2 μg/ml OPN + 0 μg/ml LM609), group 21 (2 μg/ml OPN + 1 μg/ml LM609), group 22 (2 μg/ml OPN + 2 μg/ml LM609), and group 25 (2 μg/ml OPN + 5 μg/ml LM609). PERK, Protein kinase R (PKR)-like Endoplasmic Reticulum Kinase; CHOP, C/EBP Homologous Protein; TXNIP, Thioredoxin Interacting Protein; NLRP3, Nod-like receptor family Pyrin domain containing 3; GSDMD, Gasdermin D; IL-1β, Interleukin-1 beta; Caspase 1, Cysteine-ASPartic acid proteASE 1; IL-6, Interleukin-6. Data are presented as mean ± SD. Statistical significance: ^*^ *P* < 0.05, ^**^ *P* < 0.01, ^***^ *P* < 0.001, ^****^ *P* < 0.0001.

To interpret the potential molecular mechanisms of OPN, we conducted a protein-protein interaction (PPI) analysis using the STRING database (**Supplementary Figure 4**). The results revealed significant interactions between OPN (SPP1) and cell surface receptors, particularly integrin-ανβ3, with a high confidence score of 0.999. Given the lack of specific OPN-targeting drugs, we selected LM609 (34), a monoclonal antibody against integrin-ανβ3, as an inhibitor to block OPN-mediated signaling pathways. The Western blot and RT-PCR analysis (**Figures 5A–K**) demonstrated that OPN treatment (0, 1, 2, 5 μg/ml) significantly activated the PERK-CHOP axis in cardiomyocytes and upregulated NLRP3 inflammasome-related proteins (NLRP3, GSDMD, IL-1β, IL-6), with the 2 μg/ml concentration showing peak inflammatory response. Next, we conducted intervention experiments using various concentrations of LM609 (0, 1, 2, 5 μg/ml). RT-PCR (**Figures 5L, M**) and Western blot (**Figures 5N–S**) analyses revealed that LM609 suppressed OPN-induced protein expression, effectively inhibiting both ERS and inflammasome pathway activation. These results confirmed LM609’s efficacy in blocking OPN-induced cellular stress responses, supporting OPN’s potential as a therapeutic target. It is worth mentioning that, at high LM609 concentrations (5 μg/ml), some indicators showed abnormal upregulation, possibly related to potential drug toxicity effects. These findings elucidate the molecular mechanisms mediated by OPN through integrin-ανβ3 and provide crucial evidence for developing targeted therapeutic strategies.

### Serum OPN levels correlate with NT-proBNP and show clinical significance in RVF

**Figure 6:**
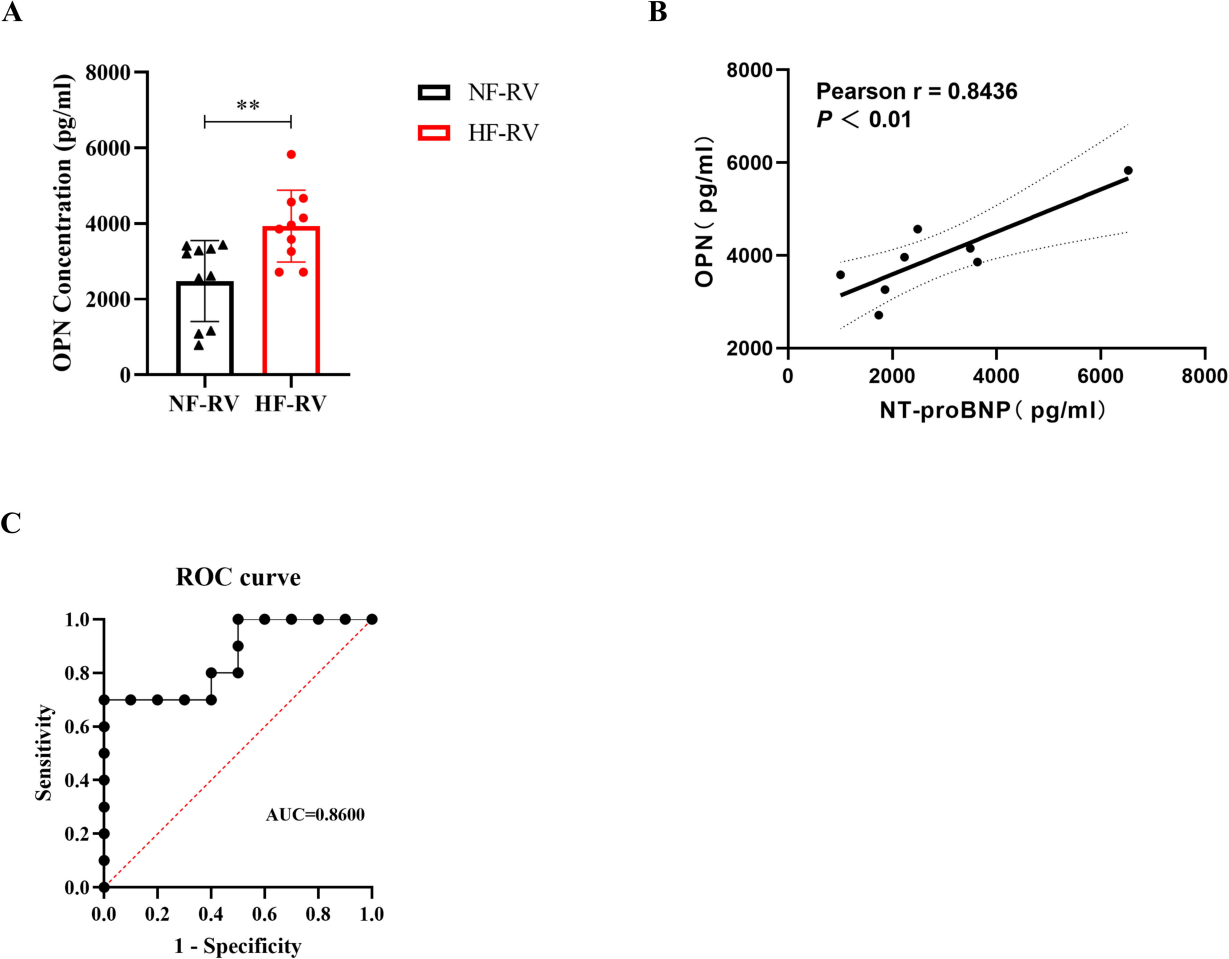
Clinical significance of serum OPN levels in right ventricular failure patients. **(A)** Serum OPN concentrations measured by ELISA in patients with normal right ventricular function (NF-RV, n=10) and pulmonary arterial hypertension-associated right heart failure (HF-RV, n=10). **(B)** Pearson correlation analysis between serum OPN and NT-proBNP levels in the HF-RV group (n=8). Solid line: linear regression fit; dashed lines: 95% confidence intervals. **(C)** Receiver Operating Characteristic (ROC) curve evaluating the diagnostic performance of serum OPN for right ventricular dysfunction. OPN, Osteopontin; NF-RV, Normal Right Ventricular Function; HF-RV, Right Heart Failure; PAH, Pulmonary Arterial Hypertension; NT-proBNP, N-terminal pro-Brain Natriuretic Peptide; ROC, Receiver Operating Characteristic; AUC, Area Under the Curve. Data presentation: Mean ± SD. Statistical significance: ^**^ *P* < 0.01.

To evaluate the clinical significance of OPN in RVF, we performed ELISA analysis on patient serum samples (**Figure 6A**). The results revealed significantly elevated serum OPN levels in RVF patients compared to healthy controls (‘HF-RV’ mean ± SD=2479.18 ± 1067.89, ‘NF-RV’ mean ± SD=3931.36 ± 951.31, *P* =0.0048). To further evaluate the clinical utility of OPN in RVF, we analyzed the correlation between serum OPN and NT-proBNP, the biomarker for heart failure (**Figure 6B**), which demonstrated a strong positive correlation (R = 0.8436, *P* =0.0085). Additionally, Receiver Operating Characteristic (ROC) curve analysis was conducted to assess the diagnostic potential of serum OPN for RVF, yielding an Area Under the Curve (AUC) of 0.8600, indicating its robust diagnostic value based on our clinical dataset. These findings validate the clinical utility of OPN in RVF.

## Discussion

**Figure 7:**
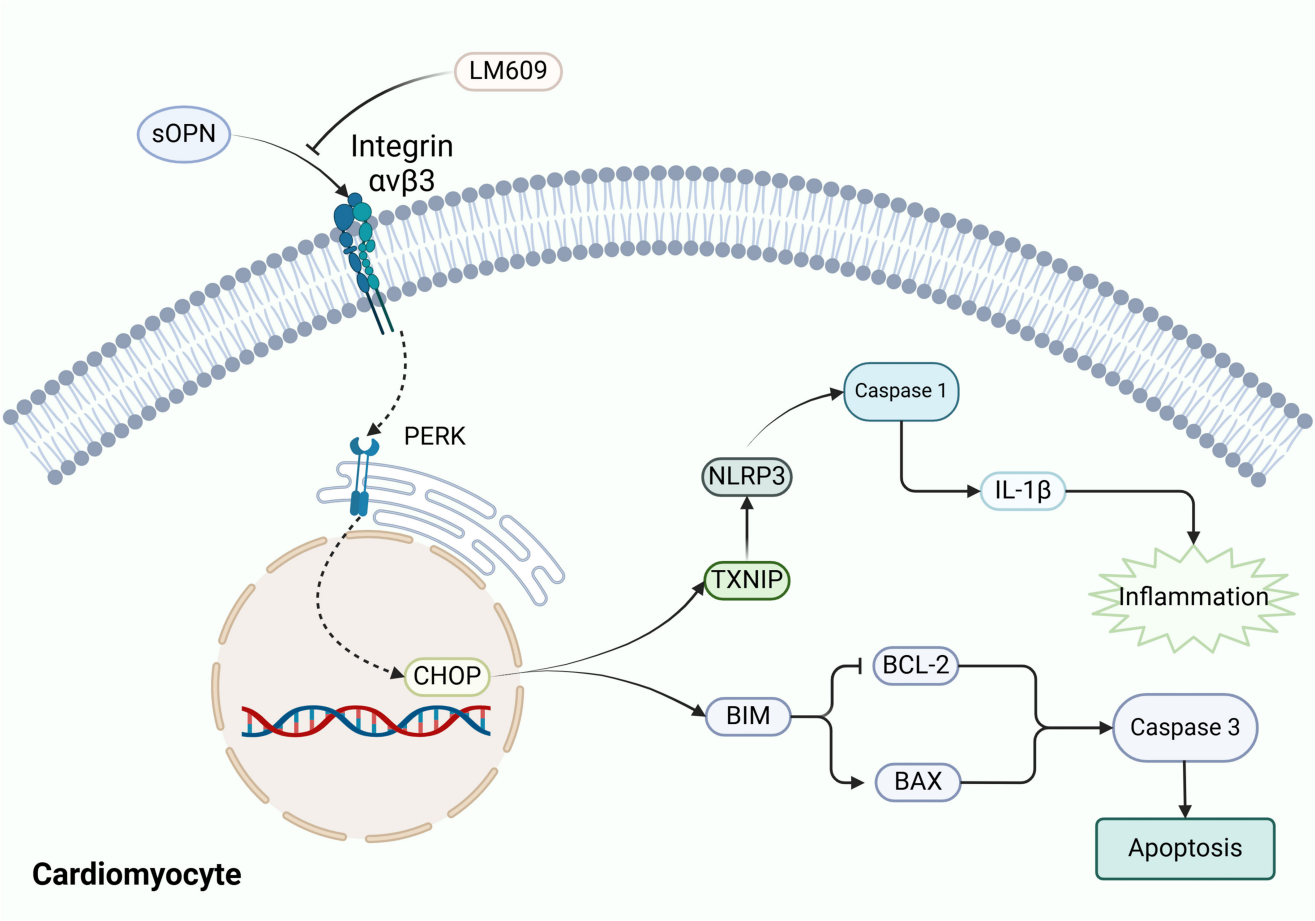
Schematic representation of OPN-mediated signaling pathways in cardiomyocytes. The diagram illustrates the proposed interaction between OPN and Integrin-ανβ3 (blocked by LM609), leading to the activation of ERS manifested as PERK and CHOP elevation. Activated ERS triggers downstream inflammatory responses (TXNIP, NLRP3, Caspase-1, IL-1β) and apoptotic pathways (BIM, BCL-2, BAX, Caspase-3). Arrows indicate hypothesized signaling cascades. OPN, Osteopontin; PERK, Protein kinase R-like ER kinase; CHOP, C/EBP Homologous Protein; TXNIP, Thioredoxin Interacting Protein; NLRP3, NOD-Like Receptor family Pyrin domain containing 3; BIM, BCL-2 Interacting Mediator of cell death; BAX, BCL2-Associated X Protein; BCL-2, B-Cell Lymphoma 2.

Recent evidence has increasingly demonstrated that inflammatory and immune responses are involved in the pathological progression of RVF (36). In this study, we performed bioinformatics analyses on GEO datasets, validated our findings using an RVF rat model, and conducted mechanistic and clinical investigations. We observed pronounced mononuclear macrophage infiltration and elevated osteopontin (OPN) expression in RV tissues of RVF patients, with OPN mediating inflammation and apoptosis via the PERK-CHOP branch of the endoplasmic reticulum stress (ERS) pathway (**Figure 7**). Serum OPN levels in RVF patients strongly correlated with NT-proBNP, suggesting its clinical significance and potential as a therapeutic target (37).

To characterize the immune regulatory mechanisms in RVF, we initially analyzed the GEO datasets (GSE161473) to compare immune-related gene expression changes and immune cell infiltration patterns between RVF and healthy controls. Our analysis identified 22 significantly differentially expressed immune-related genes and revealed a marked increase in monocyte-macrophage infiltration in the right ventricular tissue (**Figures 1**, **3**). As key players in innate immunity, macrophages maintain tissue homeostasis through phagocytosis of apoptotic cells and chemokine secretion (38). Our study further demonstrated an imbalance in the M1/M2 macrophage ratio in RVF right ventricular tissue, characterized by increased M2 macrophage expression and decreased M1 macrophage expression. This pattern resembles the inflammatory process following myocardial infarction, where M1 macrophages dominate the early inflammatory response while M2 macrophages play crucial roles in later tissue repair (39). Al-Qazazi et al. also showed significant monocyte-macrophage infiltration in PAH-induced RVF models (9). Currently, the specific mechanisms of M2 macrophages in the RVF repair phase and their relationship with disease progression remain unclear and warrant further investigation.

To validate the reliability of identified IRDEGs, we performed cross-validation analysis using the GSE120852 dataset and RNA sequencing data from RVF rat RV tissue. The SPP1 gene emerged as consistently upregulated across these datasets and was subsequently selected as our key target gene, with its encoded protein OPN designated as the focus molecule (40). Subsequent experiments confirmed significantly elevated OPN expression in the RVF model (**Figure 2**). This finding aligns with previous studies: OPN is upregulated in the left ventricular tissue of heart-failing patients with dilated cardiomyopathy (41), and involved in cardiac remodeling through the maintenance of extracellular matrix homeostasis (42). Our study extended these findings and characterized OPN upregulation in RVF.

To further investigate the underlying mechanisms, we analyzed the GSE161473 database, which revealed marked alterations in ER-related gene expression profiles in failing cardiomyocytes directing our attention toward the endoplasmic reticulum stress (ERS) pathway. *In vitro*, our experiments demonstrated that recombinant OPN protein treatment significantly induced cardiomyocyte ERS response, characterized by enhanced PERK activity and upregulation of its downstream effector CHOP (**Figures 4**, **5**). As detailed by Ren et al., activated PERK induces CHOP expression, thereby regulating cell fate in inflammatory responses and apoptosis (35). Our experimental results validated the discovery of the CHOP-driven TXNIP-NLRP3 inflammatory pathway (43) and confirmed the role of CHOP in promoting apoptosis (44). Furthermore, when we blocked integrin-ανβ3 using the neutralizing antibody LM609 (Etaracizumab), inflammatory responses were partially attenuated. Our findings align with Li et al.’s report that OPN signaling inhibition can partially reverse heart failure and improve cardiac function (45), establishing the OPN-ERS axis in RVF pathogenesis and providing insights for developing targeted therapeutic strategies.

The elucidation of OPN’s mechanistic role prompted us to explore its potential value in clinical diagnosis and prognosis assessment (**Figure 6**). While previous studies have recognized OPN’s potential as a biomarker for heart failure, these investigations primarily focused on left heart failure (46, 47). It is indicated that OPN, as a key molecule, is involved in the pathological processes of heart failure (HF), such as myocardial fibrosis and remodeling. In patients with hypertension-related heart disease, OPN is closely associated with the excessive deposition of lysyl oxidase (LOX) and insoluble collagen, which leads to left ventricular stiffness and contractile dysfunction (48). Increased OPN expression in the heart or plasma is generally negatively correlated with cardiac function (49). In heart failure with preserved ejection fraction (HFpEF), OPN exacerbates left ventricular diastolic dysfunction, while OPN blockage can improve myocardial energy metabolism and fibrosis (50). Rosenberg et al. pioneered the exploration of OPN’s utility in pulmonary hypertension (PH) patients by analyzing the correlation between serum OPN levels and right ventricular functional and morphological parameters assessed by echocardiography, establishing OPN’s preliminary role as a predictor of right ventricular dysfunction (37). To extend previous findings, we investigated the relationship between OPN and NT-proBNP, an established biomarker for heart failure, using a comprehensive analytical approach. Statistical analysis revealed a significant positive correlation between these two parameters. Furthermore, ROC curve analysis demonstrated that OPN exhibited excellent diagnostic performance, providing robust evidence for its potential clinical value in RVF.

Regarding the potential value of OPN as a specific biomarker for RVF, it is crucial to account for its association with other diseases, such as renal dysfunction (12). OPN levels are elevated in the serum of patients with acute and chronic kidney disease (51, 52), as well as in those with cirrhosis (53) and hepatocellular carcinoma (54). These elevations may arise from local or systemic immune activation. Our PAH-RVF cohort excluded patients with severe liver diseases, kidney dysfunction, and left heart failure, minimizing confounding effects. Nonetheless, the inflammatory microenvironment associated with pulmonary hypertension may partially contribute to the increased serum OPN levels observed (55). Consequently, the elevated expression of OPN observed in our study may be a consequence of the failing right ventricle and remodeled pulmonary vasculature. Additional investigations will be necessary to determine whether OPN may serve as a reliable biomarker specifically and sensitively for right heart failure.

Despite the progress made in this study, several limitations need to be addressed. First, as mentioned above, we have not experimentally determined the specific source of OPN, which limits our comprehensive understanding of its mechanistic role in RVF development. Second, this study only validated RVF secondary to PAH, without exploring RVF due to other etiologies, which somewhat limits the generalizability of our findings. Additionally, the reliance on neonatal rat cardiomyocytes in our *in vitro* experiments, while necessitated by the technical challenges of isolating and maintaining viable adult cardiomyocytes under culture conditions, may constrain the translation of findings to adult cardiac pathophysiology (56). Moreover, we cannot determine whether the observed phenomena are chamber-specific without concurrent left ventricular studies. The lack of suitable specific OPN inhibitors limited our ability to conduct mechanistic studies in animal models, consequently leaving its therapeutic potential to be verified. In terms of clinical research, the limited patient sample size may have affected the statistical significance of some results. Furthermore, we have not fully evaluated the dose-dependent cytotoxicity of the OPN inhibitor (LM609) in cellular experiments. These aspects warrant further investigation in subsequent studies.

## Conclusion

In conclusion, this study elucidates the pivotal role of osteopontin (OPN) in right ventricular failure (RVF) progression through its regulation of cardiomyocyte inflammation and apoptosis. Clinically, we discovered significantly elevated serum OPN levels in RVF patients, which positively correlate with disease severity. Our analyses revealed substantial changes in immune cell composition and inflammatory markers in RV tissues, underscoring the involvement of inflammation in RVF pathogenesis. Specifically, OPN-induced activation of the Integrin-ανβ3/PERK/CHOP pathway spurs inflammatory and apoptotic processes. The identification of this dual-pathway activation mediated by OPN highlights its potential as a promising therapeutic target for RVF. Future studies should focus on developing targeted interventions against OPN or its downstream signaling components to offer novel therapeutic strategies for patients with RVF.

## Data Availability

The datasets analyzed for this study can be found in the Gene Expression Omnibus (GEO) database: GSE161473 and GSE120852.
