# Supplementary material for: Osteopontin regulates right ventricular failure through integrin ανβ3/PERK/CHOP-dependent inflammatory and apoptotic pathways
Source: Front Immunol. 2025 May 6;16:1569210. doi: 10.3389/fimmu.2025.1569210 (PMC12088963; doi:10.3389/fimmu.2025.1569210)
Supplement: Supplementary file 1 [file DataSheet1.pdf]

Supplemental Materials

Osteopontin Regulates Right Ventricular Failure through Integrin αvβ3/PERK/CHOP-Dependent Inflammatory and Apoptotic Pathways

Xiaomei Yang[1,2†], Xuyang Wang[1,3†], Kai Li [1†], Qiming Deng[4], Yonghao Hou[1,2], Guangmin Xi[5], Kangping Lu[6,7], Zihua Liu[8], Yu Bai[2,9], Jianbo Wu[10,11,12], Jingui Yu[1], Peng Zhang[1]*

[1] Department of Anesthesiology, Qilu Hospital of Shandong University, Shandong University, Jinan, Shandong, China

[2] National Key Laboratory for Innovation and Transformation of Luobing Theory; The Key Laboratory of Cardiovascular Remodeling and Function Research, Chinese Ministry of Education, Chinese National Health Commission and Chinese Academy of Medical Sciences, Jinan, Shandong, China

[3] Department of Anesthesiology, Shandong Provincial Qian Foshan Hospital, Shandong University, Jinan, Shandong, China

[4] Hypertension Center, Beijing Anzhen Hospital, Capital Medical University, Beijing, China.

[5] College of Life Science, Qi Lu Normal University, Jinan, Shandong, China.

[6] The Second Clinical Medical School of Shandong University, Shandong University, Jinan, Shandong, China

[7] Department of Hepatobiliary Surgery, The Second Hospital of Shandong University, Shandong University, Jinan, Shandong, China

[8] Department of Cardiovascular Surgery, Qilu Hospital of Shandong University, Jinan, China.

[9] Department of Cardiology, Qilu Hospital of Shandong University, Jinan, Shandong, China

[10] Department of Anesthesiology, The First Affiliated Hospital of Shandong First Medical University, Jinan, Shandong, China

[11] Shandong Institute of Anesthesia and Respiratory Critical Care Medicine, the First Affiliated Hospital of Shandong First Medical University, Jinan, Shandong, China

[12] Shandong Provincial Clinical Research Center for Anesthesiology, the First Affiliated Hospital of Shandong First Medical University, Jinan, Shandong, 250014, China

Supplement Methods

RV pressure measuring

Due to the absence of significant pulmonary valve disease in rodent models, right ventricular systolic pressure (RVSP) can serve as a reliable surrogate measure for pulmonary artery systolic pressure(1). Before the end of the 4-5 weeks experimental period, right ventricular pressure curves were recorded during anesthesia. All experimental animals were anesthetized via intraperitoneal injection of 50 mg/kg pentobarbital sodium. Using the BL-420N biological signal acquisition and analysis system, the right ventricular pressure was measured and recorded via a trans-diaphragmatic puncture method. Specifically, anesthetized rats were placed supine on the operation table, and their abdomens were prepared and disinfected. An incision of 2-3 cm was made from the xiphoid process along the lower edge of the costal arch. By gently lifting the xiphoid process, the beating heart on the diaphragm was exposed, indicating the puncture site. A puncture needle connected to the instrument and heparinized saline was inserted horizontally into the center of the beating area to measure and record the right ventricular pressure. The recorded pressure curves were then statistically analyzed using the corresponding software.

Supplement Result

In the gene expression analysis from RV tissues of end-stage heart failure patients with biventricular failure (***Supplement Figure 2***), only CXCL14 showed significant differential expression ($P < 0.0001$). Notably, although SPP1 showed an upward trend in the HF-RV group, it did not reach statistical significance due to high intergroup variability, while RNASE2 showed neither an upward trend nor significant difference.

Supplemental figures

Supplement Figure 1. Workflow of bioinformatic analysis.

The flowchart illustrates the comprehensive bioinformatic analysis pipeline used in this study. Starting with data acquisition from GSE161473, differential expression analysis was performed using the limma package (|log2 FC| > 2, $P < 0.05$). Differentially expressed genes were matched with the ImmPort database to identify immune-related DEGs (IRDEGs). Downstream analyses included: (1) Visualization using ggplot2 and heatmap packages; (2) Cross-validation with GSE129823 and rat RV transcriptome data through SPPI analysis; (3) Enrichment analysis using ClusterProfiler for GO and KEGG pathways; (4) Gene Set Enrichment Analysis (GSEA); (5) Immune cell infiltration analysis using CIBERSORT; and (6) Correlation analysis using Corrplot.

Supplement Figure 2. Differential Expression Analysis of IRDEGs between NF-RV and HF-RV Groups

Box plots comparing relative expression levels of IRDEGs (*CXCL14*, *TRIM22*, *SPP1*, *TAP2*, *RNASE2,* and others) identified from the GSE120852 database. Groups: NF-RV (Normal Right Ventricular Function) and HF-RV (Right Heart Failure). **Abbreviations:** IRDEGs, Immune-Related Differentially Expressed Genes; NF-RV, Normal Right Ventricular Function; HF-RV, Right Heart Failure. Statistical analysis: Two-way ANOVA. n=5 per group. Significance: $^{***}P < 0.0001$

Supplement Figure 3. Immunohistochemical Staining of CD14 in Control and RVF Cardiac Tissues

(A) Representative images of CD14 staining in Control and RVF groups. Upper panels: Low magnification (scale bar: 20 μm); Lower panels: High magnification (scale bar: 20 μm). (B) Quantitative analysis of CD14 expression by integrated optical density (IOD). n = 3 rats/group. Abbreviations: RVF, Right Ventricular Failure; IOD, Integrated Optical Density. Data presentation: Mean ± SD. Statistical significance: $P < 0.05$ vs. Control.

Supplement Figure 4. Construction of protein-protein interaction (PPI) network for SPP1(OPN)

The protein-protein interaction (PPI) network was constructed using the STRING database and visualized using PPI network software. Based on network topology parameters, several genes showing high connectivity to SPP1 were identified, including integrin family members (ITGA9, ITGAV, ITGB1, and ITGB3), matrix metalloproteinases (MMP3 and MMP7), CD44,

fibronectin 1 (FN1), adaptor-related protein complex 4 subunit mu 1 (AP4M1), and ras homolog family member A (RHOA). Among them, integrins (including ITGAV, ITGB1, and ITGB3) and CD44 serve as receptors for SPP1(2). MMP3 is a protease capable of cleaving SPP1 at specific sites(3). This interaction network suggests the complex regulatory relationships between SPP1 and its interaction partners.

Supplement Figure 5. Development and Characterization of MCT-Induced RVF Model
(A) Schematic of the experimental protocol. Male Sprague-Dawley (SD) rats (250-300 g) received intraperitoneal injections of saline (Control group) or monocrotaline (MCT, 60 mg/kg; RVF group), followed by 4-5 weeks of monitoring. (B) Representative right ventricular pressure waveforms (Control vs. RVF). (C) Right ventricular systolic pressure (RVSP) measurements. n = 5 rats/group. (D) Body weight trajectories over 35 days post-injection. n = 5 rats/group. (E) Fulton index (right ventricular weight / [left ventricular + septum weight]). n = 5 rats/group. **Abbreviations**: RVF, Right Ventricular Failure; MCT, Monocrotaline; RVSP, Right Ventricular Systolic Pressure. Data presentation: Mean ± SD. Statistical analysis: Unpaired Student's *t*-test. Significance: $^{*}P < 0.05$, $^{***}P < 0.001$, $^{****}P < 0.0001$ *vs.* Control.

Supplementary Table 1. The Information on Immune-Related Differentially Expressed Genes (IRDEGs)

Abbreviation	Full Title	Function
OPN (SPP1)	Osteopontin	Binds tightly to hydroxyapatite. Appears to form an integral part of the mineralized matrix. Probably important to cell-matrix interaction. It enhances the role of macrophages and T cells during inflammation. (314 aa)
RNASE2	Ribonuclease A2	This is a non-secretory ribonuclease. It is a pyrimidine-specific nuclease with a slight preference for U. Cytotoxin and helminthotoxin. Selectively chemotactic for dendritic cells. Possesses a wide variety of biological activities and belongs to the pancreatic ribonuclease family. (161 aa)
HLA-DRA	HLA class II histocompatibility antigen, DR alpha chain	Bind peptides derived from antigens that access the endocytic route of antigen-presenting cells (APC) and present them on the cell surface for recognition by the CD4 T-cells. The peptide binding cleft accommodates peptides of 10-30 residues. The peptides presented by MHC class II molecules are generated mostly by the degradation of proteins that access the endocytic route, where they are processed by lysosomal proteases and other hydrolases. (254 aa)
CD74	HLA class II histocompatibility antigen gamma chain	Plays a critical role in MHC class II antigen processing by stabilizing peptide-free class II alpha/beta heterodimers in a complex soon after their synthesis and directing transport of the complex from the endoplasmic reticulum to the endosomal/lysosomal system where the antigen processing and binding of antigenic

		peptides to MHC class II takes place. Serves as cell surface receptor for the cytokine MIF. (296 aa)
CX3CL1	Processed fractalkine	Acts as a ligand for both CX3CR1 and integrins. Binds to CX3CR1. Binds to integrins ITGAV: ITGB3 and ITGA4:ITGB1. Can activate integrins in both a CX3CR1-dependent and CX3CR1-independent manner. In the presence of CX3CR1, activates integrins by binding to the classical ligand-binding site (site 1) integrins. In the absence of CX3CR1, binds to a second site (site 2) in integrins which is distinct from site 1 and enhances the binding of other integrin ligands to site 1. The soluble form is chemotactic for T-cells and monocytes and not for neutrophils. (397 aa)
TRIM22	Tripartite Motif Containing 22	E3 ubiquitin-protein ligase TRIM22. Interferon-induced antiviral protein involved in cell innate immunity. The antiviral activity could in part be mediated by TRIM22-dependent ubiquitination of viral proteins. Plays a role in restricting the replication of HIV-1, encephalomyocarditis virus (EMCV), and hepatitis B virus (HBV). Acts as a transcriptional repressor of HBV core promoter. May have E3 ubiquitin-protein ligase activity. (498 aa)
IDO1	Indoleamine 2,3-dioxygenase 1	Catalyzes the first and rate-limiting step of the catabolism of the essential amino acid tryptophan along the kynurenine pathway. Involved in the peripheral immune tolerance, contributing to maintaining homeostasis by preventing autoimmunity or immunopathology that would result from uncontrolled and overreacting immune responses.

		Tryptophan shortage inhibits T lymphocyte division and accumulation of tryptophan catabolites induces T-cell apoptosis and differentiation of regulatory T-cells. Acts as a suppressor of anti-tumor immunity. (403 aa)
HLA-F	HLA class I histocompatibility antigen, alpha chain F	Non-classical major histocompatibility class Ib molecule is postulated to play a role in immune surveillance, immune tolerance, and inflammation. Functions in two forms, as a heterotrimeric complex with B2M/beta-2 microglobulin and a peptide (peptide-bound HLA-F-B2M) and as an open conformer (OC) devoid of peptide and B2M (peptide-free OC). In complex with B2M, presents non-canonical self-peptides carrying post-translational modifications, particularly phosphorylated self-peptides. (442 aa)
TAP2	Antigen peptide transporter 2	Involved in the transport of antigens from the cytoplasm to the endoplasmic reticulum for association with MHC class I molecules. Also acts as a molecular scaffold for the final stage of MHC class I folding, namely the binding of peptides. Nascent MHC class I molecules are associated with TAP via tapasin. Inhibited by the covalent attachment of herpes simplex virus ICP47 protein, which blocks the peptide-binding site of TAP. (686 aa)
HLA-DOA	HLA class II histocompatibility antigen, DO alpha chain	The important modulator in the HLA class II-restricted antigen presentation pathway by interaction with the HLA-DM molecule in B- cells. Modifies peptide exchange activity of HLA-DM. (250 aa)
CXCL9	C-X-C motif chemokine 9	Cytokine affects the growth, movement, or activation state of cells that participate in immune and inflammatory response.

		Chemotactic for activated T-cells. Binds to CXCR3; Belongs to the intercrine alpha (chemokine CxC) family. (125 aa)
ISG20	Interferon-stimulated gene 20 kDa protein	Interferon-induced antiviral exoribonuclease acts on single-stranded RNA and has minor activity towards single-stranded DNA. Exhibits antiviral activity against RNA viruses including hepatitis C virus (HCV), hepatitis A virus (HAV), and yellow fever virus (YFV) in an exonuclease-dependent manner. May also play additional roles in the maturation of snRNAs and rRNAs, and in ribosome biogenesis. (181 aa)
FGF12	Fibroblast growth factor 12	Involved in nervous system development and function. Involved in the positive regulation of voltage-gated sodium channel activity. Promotes neuronal excitability by elevating the voltage dependence of neuronal sodium channel SCN8A fast inactivation. (243 aa)
CXCL14	C-X-C motif chemokine 14	Potent chemoattractant for neutrophils, and weaker for dendritic cells. Not chemotactic for T-cells, B-cells, monocytes, natural killer cells, or granulocytes. Does not inhibit the proliferation of myeloid progenitors in colony formation assays. (111 aa)
ADM	Adrenomedullin	AM and PAMP are potent hypotensive and vasodilatator agents. Numerous actions have been reported most related to the physiological control of fluid and electrolyte homeostasis. In the kidney, AM is diuretic and natriuretic, and both am and pamp inhibit aldosterone secretion by direct adrenal actions. In the pituitary gland, both peptides at physiologically

		relevant doses inhibit basal ACTH secretion. (185 aa)
AREG	Amphiregulin	Ligand of the EGF receptor/EGFR. Autocrine growth factor as well as a mitogen for a broad range of target cells including astrocytes, Schwann cells, and fibroblasts; Belongs to the amphiregulin family. (252 aa)
CCL14	C-C motif chemokine 14	Has weak activities on human monocytes and acts via receptors that also recognize MIP-1 alpha. It induces intracellular Ca^{2+} changes and enzyme release, but no chemotaxis, at concentrations of 100-1,000 nM, and is inactive on T-lymphocytes, neutrophils, and eosinophil leukocytes. Enhances the proliferation of CD34 myeloid progenitor cells. The processed form HCC-1(9-74) is a chemotactic factor that attracts monocytes, eosinophils, and T-cells and is a ligand for CCR1, CCR3, and CCR5; Belongs to the intercrine beta (chemokine CC) family. (109 aa)
TRAC	Nuclear receptor corepressor 2	Transcriptional corepressor. Mediates the transcriptional repression activity of some nuclear receptors by promoting chromatin condensation, thus preventing access to the basal transcription. Isoform 1 and isoform 4 have different affinities for different nuclear receptors. Involved in the regulation BCL6-dependent of germinal center (GC) reactions, mainly through the control of the GC B-cells proliferation and survival. (2514 aa)
IGHM	Immunoglobulin heavy constant mu	The IGHM molecule, known as the Immunoglobulin Heavy Chain Mu, plays a crucial role in the immune system. It is a key component of IgM antibodies, which

		are the first antibodies produced during the primary immune response, rapidly identifying and neutralizing pathogens. IGHM also forms part of the B cell receptor (BCR) on B cells, enabling the recognition and binding of antigens, leading to B cell activation, proliferation, and differentiation into antibody-producing cells. Additionally, IgM antibodies are highly effective in activating the complement system via the classical pathway, enhancing pathogen clearance. As a pentamer, IgM efficiently agglutinates pathogens, facilitating their recognition and elimination by the immune system. IGHM is essential for the rapid and effective initial defense against infections.
IGHA1	Immunoglobulin heavy constant alpha 1	The IGHA1 molecule, known as Immunoglobulin Heavy Chain Alpha 1, is a critical component of the immune system. It is a major part of IgA1 antibodies, which play a significant role in mucosal immunity. IgA1 antibodies are primarily found in mucous membranes lining the respiratory, gastrointestinal, and genitourinary tracts, where they help protect against infections by neutralizing pathogens and preventing their adherence to epithelial cells. IGHA1 also forms part of the secretory IgA (sIgA) found in bodily secretions such as saliva, tears, and breast milk, providing an essential immune defense for infants and mucosal surfaces. Additionally, IgA1 antibodies can mediate immune responses through processes such as antibody-dependent cellular cytotoxicity (ADCC) and the activation of immune cells, contributing to the overall immune

		surveillance and protection of the body's mucosal surfaces.
IGHG1	Immunoglobulin heavy constant gamma 1 (G1m marker)	The IGHG1 molecule, known as Immunoglobulin Heavy Chain Gamma 1, is a crucial component of the immune system. It forms part of IgG1 antibodies, the most abundant subclass in human serum. IgG1 antibodies play a pivotal role in immune defense by neutralizing pathogens and toxins, opsonizing pathogens to enhance their uptake and destruction by phagocytes, and activating the complement system to further facilitate pathogen elimination. Additionally, IgG1 antibodies mediate antibody-dependent cellular cytotoxicity (ADCC) by recruiting natural killer (NK) cells to target and destroy infected or malignant cells. The IGHG1 molecule is essential for providing robust and versatile immune protection, as well as contributing to immune memory by enabling the body to respond more effectively to repeated exposures to the same pathogen.
IGKC	Immunoglobulin kappa constant	The IGKC molecule, known as Immunoglobulin Kappa Constant, is an essential component of the immune system. It is part of the kappa light chain of immunoglobulins, which, along with heavy chains, form the structure of antibodies. These antibodies are crucial for the adaptive immune response, as they help recognize and bind to specific antigens on pathogens. The kappa light chain, including IGKC, plays a critical role in the antigen-binding process, contributing to the diversity and specificity of the immune response. By forming a part of various

	immunoglobulin classes, the IGKC molecule helps in neutralizing pathogens, facilitating their destruction, and supporting immune system functions such as opsonization and complement activation.

Note: The functional annotations were retrieved from the STRING database (https://string-db.org/).

Supplementary Table 2. RT-qPCR Primers

Gene Name	Species	F（5’- 3’）	R（5’- 3’）	Size (bp)
SPP1 (OPN)	Rat	CCAGCCAAGGACCAA CTACA	CTGCCAAACTCAGCC ACTTTC	100
PERK	Rat	AGTGGACGGCGAT GATGAG	GCTGCTGGAGTGCTT GAAC	211
CHOP	Rat	ATGTTGAAGATGAG CGGGTGG	CGGTTTCTGCTTTCA GGTGTG	117
GRP78/BiP	Rat	GTGACCTGGTTCTG CTTGATG	CACCTTCGTAGACCT TGATTGTTA	171
ATF6	Rat	GCCTGCTGTGGTTC AACTTC	TCATACGCTGCTGTC TCCTC	230
IRE1	Rat	GACGGACAGAATA CACCATCAC	CCACCACAGGAGAG GCATAG	213
XBP1	Rat	TCCGCAGCACTCA GACTAC	GTTCCTCCAGATTAG CAGACTC	198
IL-6	Rat	TCCTACCCCAACTT CCAATGC	GGTTTGCCGAGTAGA CCTCAT	139
IL-1β	Rat	GACAGAACATAAG CCAACAAGTG	ACACAGGACAGGTAT AGATTCTTC	118

Abbreviations: ATF6: activating transcription factor 6, BiP: binding immunoglobulin protein, CHOP: C/EBP homologous protein, GRP78: glucose-regulated protein 78, IL-1β: interleukin 1 beta, IL-6: interleukin 6, IRE1: inositol-requiring enzyme 1, OPN: osteopontin, PERK: protein kinase R-like endoplasmic reticulum kinase, SPP1: secreted phosphoprotein 1, XBP1: X-box binding protein 1.

Supplementary Table 3. Characteristics of the patients included in the discovery set

	Right Heart Failure	Control	P
**N**	30	30	
**Clinical Characteristics**			
Age, median (IQR), year	55.000[43.000,60.000]	59.000[54.000,62.000]	0.065
Male gender, *n* (%)	17(56.7)	16(53.3)	1.000
Weight, mean(±SD), Kg	61.200±5.455	71.100±10.014	0.018
Height, mean(±SD), m	1.675±0.055	/	/
BMI, mean(±SD)	21.916±2.720	/	/
SBP, mean(±SD), mmHg	102.900±15.802	137.000±10.909	<0.001
DBP, mean(±SD), mmHg	66.400±6.406	81.700±8.149	<0.001
Heart rate, mean(±SD), beats/min	85.700±12.602	77.100±7.409	0.099
**Risk factors**			
Smoking, *n* (%)	12(40.000)	15(50.000)	0.606
Alcohol use, *n* (%)	1(3.333)	3(1.000)	1.000
Hypertension, *n* (%)	0(0.000)	6(20.000)	
Coronary Heart Disease, *n* (%)	1(3.333)	1(3.333)	1.000
Diabetes, *n* (%)	0(0.000)	1(3.333)	
COPD, *n* (%)	0(0.000)	0(0.000)	
Respiratory Failure, *n* (%)	0(0.000)	0(0.000)	
Cerebral Infarction, *n* (%)	0(0.000)	0(0.000)	
Pneumonia, *n* (%)	0(0.000)	0(0.000)	
**Drug use**			
Diuretics, *n* (%)	0(00.000)	0(0.000)	
Beta bloker, *n* (%)	1(3.333)	1(3.333)	1.000
CCB, *n* (%)	0(0.000)	1(3.333)	
ACEI, *n* (%)	0(0.000)	1(3.333)	
ARB, *n* (%)	0(0.000)	0(0.000)	
Statins, *n* (%)	0(0.000)	0(0.000)	
Anti diabetic, *n* (%)	0(0.000)	1(3.333)	

**Laboratory Examination**			
WBC, mean(±SD), 10^9/L	5.198±1.192	6.403±1.425	0.067
NEU, mean(±SD), 10^9/L	3.100±1.017	3.460±1.187	0.498
LYM, mean±SD, 10^9/L	1.434±0.473	2.397±0.720	0.004
EOS, median (IQR), 10^9/L	0.060[0.040,0.160]	0.090[0.060,0.180]	0.402
BAS, median (IQR), 10^9/L	0.020[0.020,0.060]	0.020[0.020,0.030]	0.473
MON, median (IQR), 10^9/L	0.450[0.440,0.500]	0.340[0.280,0.450]	0.063
RBC, mean±SD, 10^12/L	4.539±0.662	4.763±0.358	0.384
HGB, mean±SD, G/L	127.700±20.165	145.200±10.274	0.032
PLT,mean(±SD), 10^9/L	189.800±53.018	271.600±67.905	0.011
ALT, median (IQR), U/L	16.000[13.000,19.000]	20.000[15.000,23.000]	0.160
AST, mean(±SD), U/L	18.333±3.559	30.100±25.560	0.213
GLDH, mean(±SD), U/L	4.856±7.181	12.900±24.853	0.388
GGT, mean(±SD), U/L	44.667±28.103	39.400±27.112	0.699
AKP, median (IQR), U/L	86.000[49.000,115.000]	88.000[78.000,94.000]	0.850
ADA, mean(±SD), U/L	11.667±2.789	9.600±2.691	0.139
TBIL, median (IQR), umol/l	20.900[7.500,30.400]	9.000[6.900,11.800]	0.054
DBIL, mean(±SD), umol/l	7.189±3.608	3.350±0.854	0.017
IBIL, mean(±SD), umol/l	13.122±6.953	6.080±1.723	0.021
PA, mean(±SD), mg/dl	19.644±3.984	25.620±3.048	0.003
TP, mean(±SD), g/l	70.210±7.428	72.860±4.744	0.379
ALB, mean(±SD), g/l	42.430±4.826	45.730±2.625	0.088
GLB, mean(±SD), g/l	26.433±3.524	27.130±4.086	0.713
A/G, mean(±SD)	1.621±0.251	1.726±0.293	0.441
Cho,mean±SD, mmol/l	3.800±0.808	5.189±0.785	0.005

HDL-C,mean±SD, mmol/l	0.997±0.218	1.366±0.314	0.023
LDL-C,mean±SD, mmol/l	2.283±0.531	3.006±0.721	0.051
sdLDL, mean(±SD), mmol/l	0.445±0.201	0.963±0.509	0.043
apoA1, mean(±SD), G/L	1.212±0.126	1.608±0.114	0.004
apoB,mean±SD, G/L	0.848±0.215	1.030±0.301	0.383
TG, mean(±SD), mmol/l	1.434±0.631	1.944±0.940	0.258
LPa, mean(±SD), nmol/l	41.080±12.499	21.525±19.298	0.150
Hcy,mean(±SD), umol/l	15.212±5.494	11.730±4.369	0.177
NEFA,mean±SD,umol/dl	52.886±9.987	51.170±25.652	0.877
GLU,mean±SD, mmol/l	4.555±0.553	5.475±0.889	0.017
BUN, median (IQR), mmol/l	5.200[3.900,6.700]	4.700[4.300,5.800]	0.762
Cr,median (IQR), umol/l	65.000[56.000,107.000]	56.000[54.000,69.000]	0.427
Cys-C, mean(±SD), mg/l	1.033±0.175	0.836±0.090	0.015
UA,mean±SD, umol/l	385.400±119.841	330.800±88.811	0.287
CK,mean(±SD), U/L	61.833±17.771	85.000±34.125	0.186
hs-CTNI,mean(±SD), ng/l	11.696±6.645	1.780±0.571	0.011
CK-MB, mean(±SD), ng/ml	1.457±0.616	1.200±0.555	0.443
LDH, mean(±SD), U/L	220.286±42.280	198.900±27.024	0.251
IMA,mean(±SD), U/ml	72.767±8.092	69.488±5.088	0.407
K^+,mean(±SD), mmol/l	4.216±0.392	4.282±0.291	0.690
Na^+, mean(±SD), mmol/l	137.600±3.527	141.900±1.814	0.004
Cl^-, median (IQR), mmol/l	106.000[100.000,107.000]	106.000[105.000,107.000]	0.731
Ca^{2+}, mean(±SD), mmol/l	2.265±0.074	2.349±0.072	0.025
Mg^{2+}, median (IQR), mmol/l	0.870[0.850,0.960]	0.880[0.860,0.920]	0.939
DD-i, mean(±SD), ug/ml	0.453±0.214	0.580±0.390	0.406
**Echocardiography**			
LVEF,median[IQR]	0.600[0.600,0.600]	0.610[0.600,0.660]	0.030

IVST,mean(±SD),cm	0.830±0.127	1.040±0.111	0.002
LVPWT, median [IQR], cm	0.900[0.800,1.000]	0.900[0.900,1.000]	0.496
LVDd, mean(±SD), cm	3.640±0.531	4.180±0.790	0.106
PASP, median [IQR], mmHg	61.000[56.000,84.000]	25.000[20.000,26.000]	<0.001
LAmm, mean(±SD), mm	28.900±2.879	34.200±3.894	0.004
RV, median [IQR], mm	35.000[27.000,44.000]	23.000[23.000,25.000]	0.003
AAO, mean(±SD), mm	27.600±3.137	31.800±1.600	0.003
PA, median [IQR], mm	31.000[29.000,34.000]	22.000[21.000,24.000]	<0.001

Abbreviations: AAO: Ascending Aorta; ACEI: Angiotensin-Converting Enzyme Inhibitor; ADA: Adenosine Deaminase; AKP: Alkaline Phosphatase; ALB: Albumin; ALT: Alanine Aminotransferase; apoA1: Apolipoprotein A1; apoB: Apolipoprotein B; ARB: Angiotensin Receptor Blocker; AST: Aspartate Aminotransferase; BAS: Basophils; BMI: Body Mass Index; BUN: Blood Urea Nitrogen; CCB: Calcium Channel Blocker; CK: Creatine Kinase; CK-MB: Creatine Kinase-MB; COPD: Chronic Obstructive Pulmonary Disease; Cr: Creatinine; Cys-C: Cystatin C; DBIL: Direct Bilirubin; DBP: Diastolic Blood Pressure; DD-i: D-Dimer; EOS: Eosinophils; GGT: Gamma-Glutamyl Transferase; GLB: Globulin; GLDH: Glutamate Dehydrogenase; GLU: Glucose; HGB: Hemoglobin; Hcy: Homocysteine; HDL-C: High-Density Lipoprotein Cholesterol; hs-CTNI: High-Sensitivity Cardiac Troponin I; IBIL: Indirect Bilirubin; IMA: Ischemia Modified Albumin; IVST: Interventricular Septum Thickness; LA: Left Atrium; LDH: Lactate Dehydrogenase; LDL-C: Low-Density Lipoprotein Cholesterol; LPa: Lipoprotein(a); LVEF: Left Ventricular Ejection Fraction; LVDd: Left Ventricular End-Diastolic Diameter; LVPWT: Left Ventricular Posterior Wall Thickness; LYM: Lymphocytes; MON: Monocytes; NEU: Neutrophils; NEFA: Non-Esterified Fatty Acids; PA: Pulmonary Artery/Prealbumin; PASP: Pulmonary Artery Systolic Pressure; PLT: Platelets; RBC: Red Blood Cells; RV: Right Ventricle; SBP: Systolic Blood Pressure; sdLDL: Small Dense Low-Density Lipoprotein; TBIL: Total Bilirubin; TG: Triglycerides; TP: Total Protein; UA: Uric Acid; WBC: White Blood Cells.

Supplementary Reference

1. Song Y, Jones JE, Beppu H, Keaney JF, Jr., Loscalzo J, Zhang YY. Increased Susceptibility to Pulmonary Hypertension in Heterozygous Bmpr2-Mutant Mice. *Circulation* (2005) 112(4):553-62. Epub 20050718. doi: 10.1161/CIRCULATIONAHA.104.492488.
2. Icer MA, Gezmen-Karadag M. The Multiple Functions and Mechanisms of Osteopontin. *Clinical Biochemistry* (2018) 59:17-24. doi: 10.1016/j.clinbiochem.2018.07.003.
3. Mamazhakypov A, Sartmyrzaeva M, Sarybaev AS, Schermuly R, Sydykov A. Clinical and Molecular Implications of Osteopontin in Heart Failure. *Curr Issues Mol Biol* (2022) 44(8):3573-97. Epub 20220811. doi: 10.3390/cimb44080245.
