# Supplementary material for: Osteopontin regulates right ventricular failure through integrin ανβ3/PERK/CHOP-dependent inflammatory and apoptotic pathways
Source: Front Immunol. 2025 May 6;16:1569210. doi: 10.3389/fimmu.2025.1569210 (PMC12088963; doi:10.3389/fimmu.2025.1569210)
Supplement: Supplementary file 2 [file Presentation1.pptx]

## Slide 1
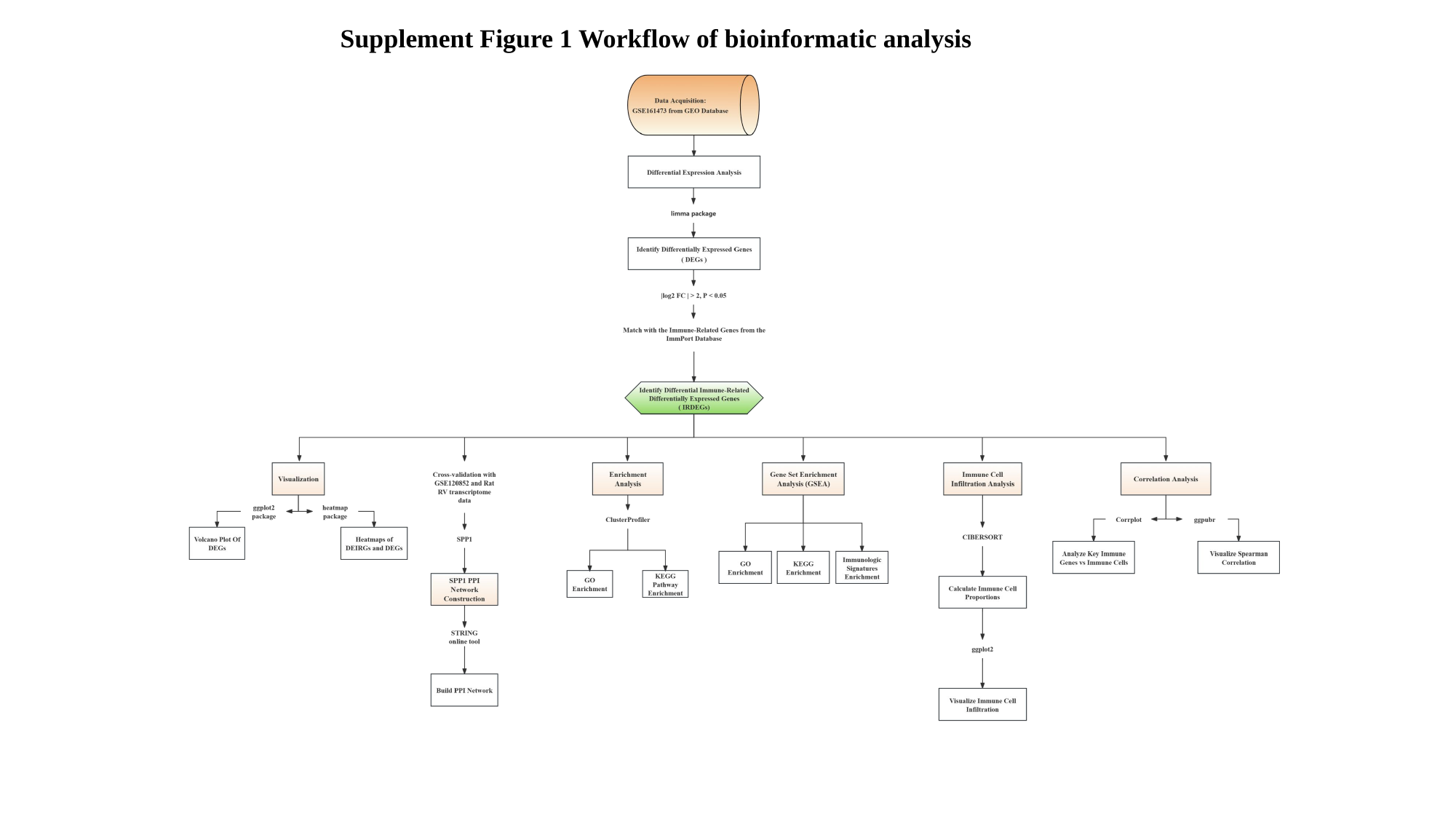

Supplement Figure 1 Workflow of bioinformatic analysis

## Slide 2
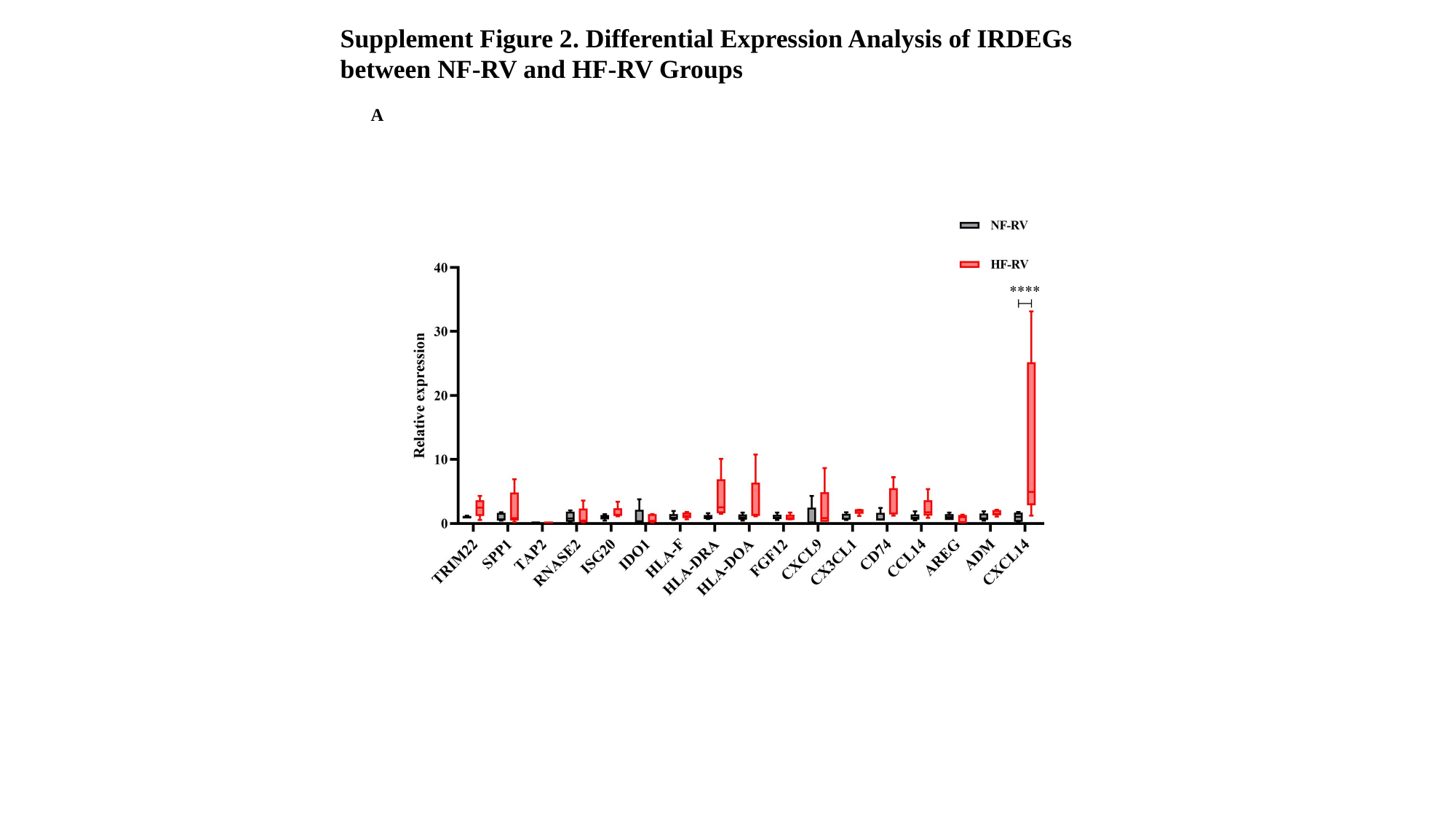

Supplement Figure 2. Differential Expression Analysis of IRDEGs between NF-RV and HF-RV Groups
A

## Slide 3
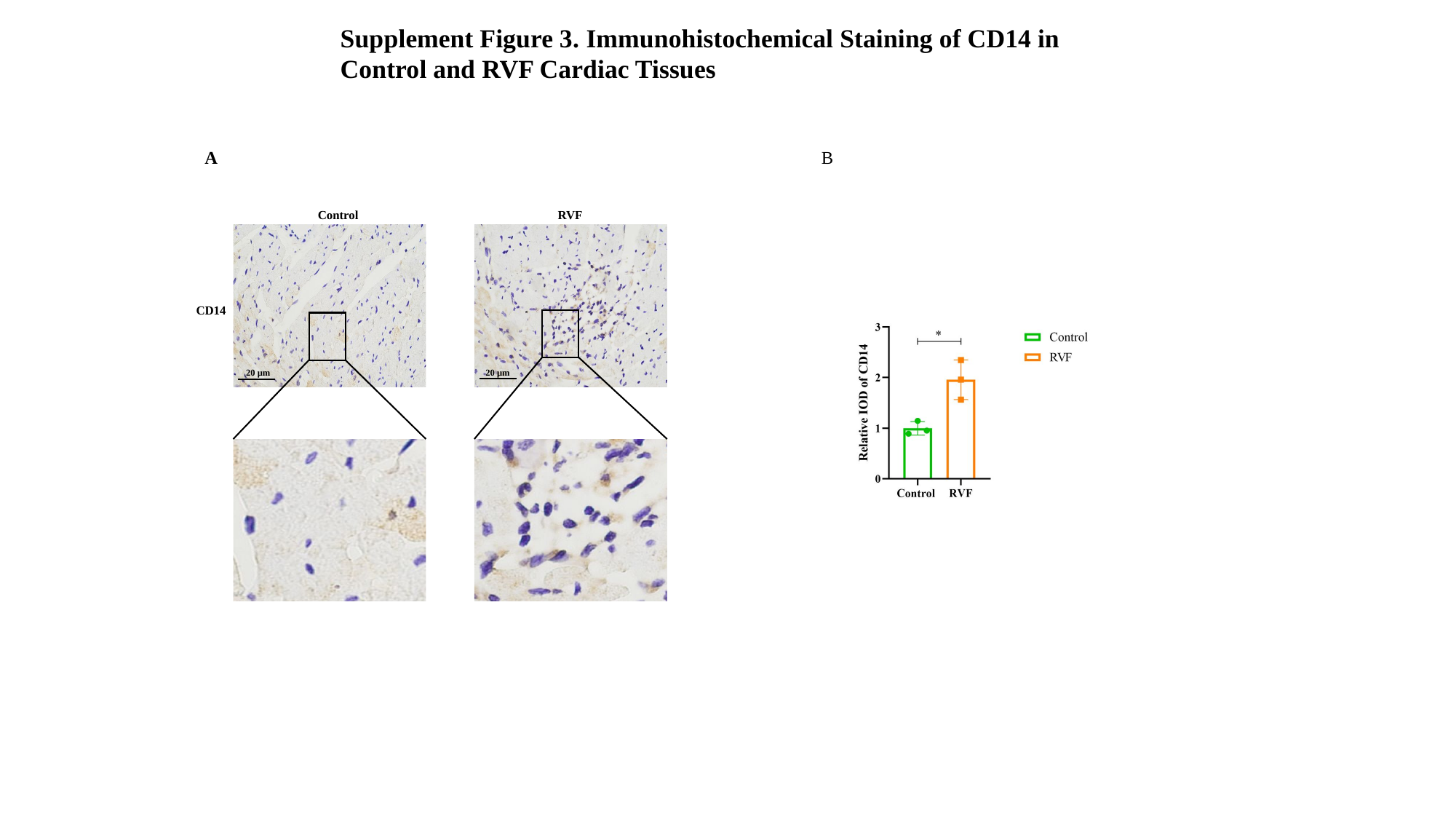

Supplement Figure 3. Immunohistochemical Staining of CD14 in Control and RVF Cardiac Tissues
A
B
Control
RVF
CD14
20 μm
20 μm

## Slide 4
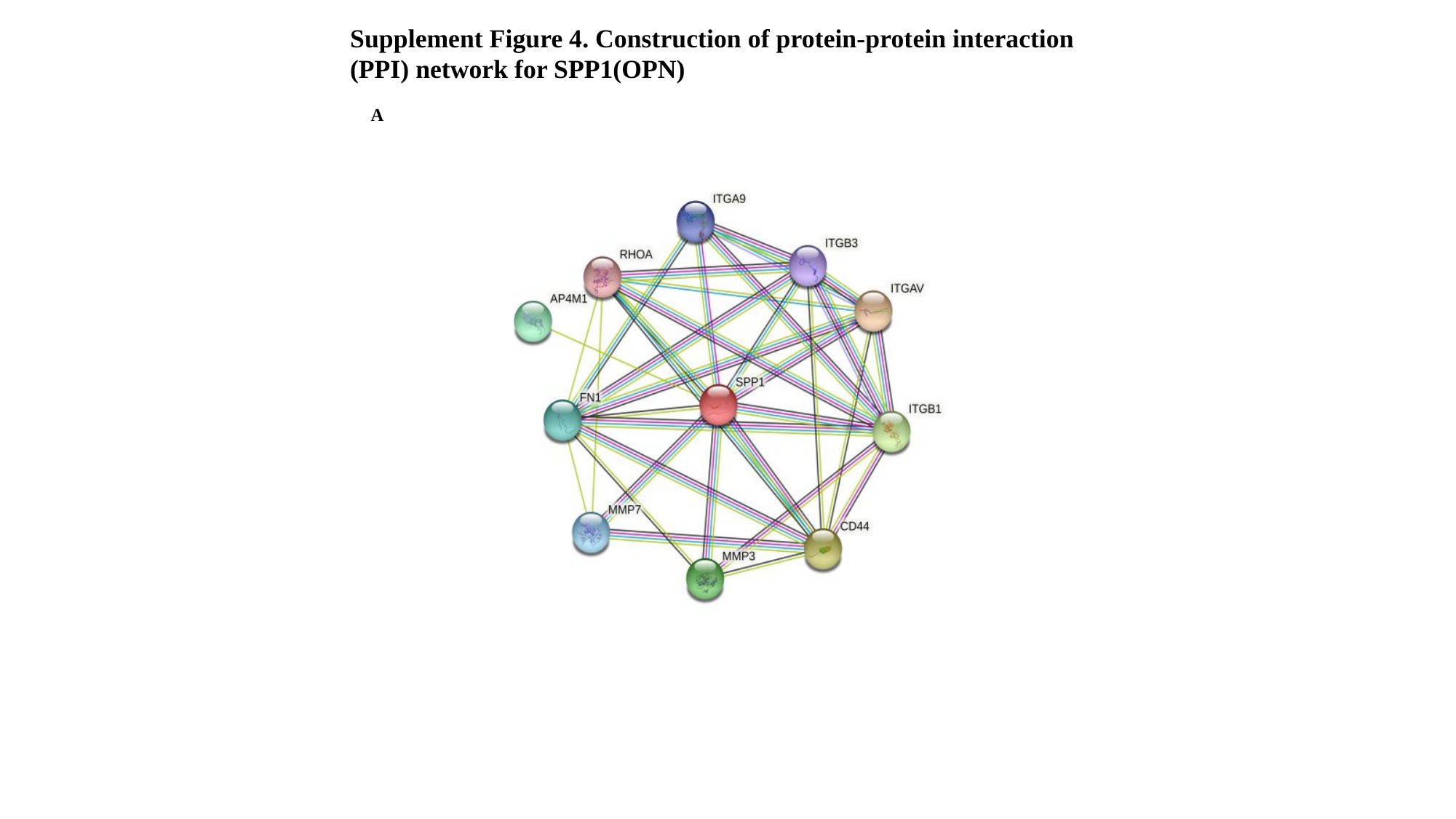

Supplement Figure 4. Construction of protein-protein interaction (PPI) network for SPP1(OPN)
A

## Slide 5
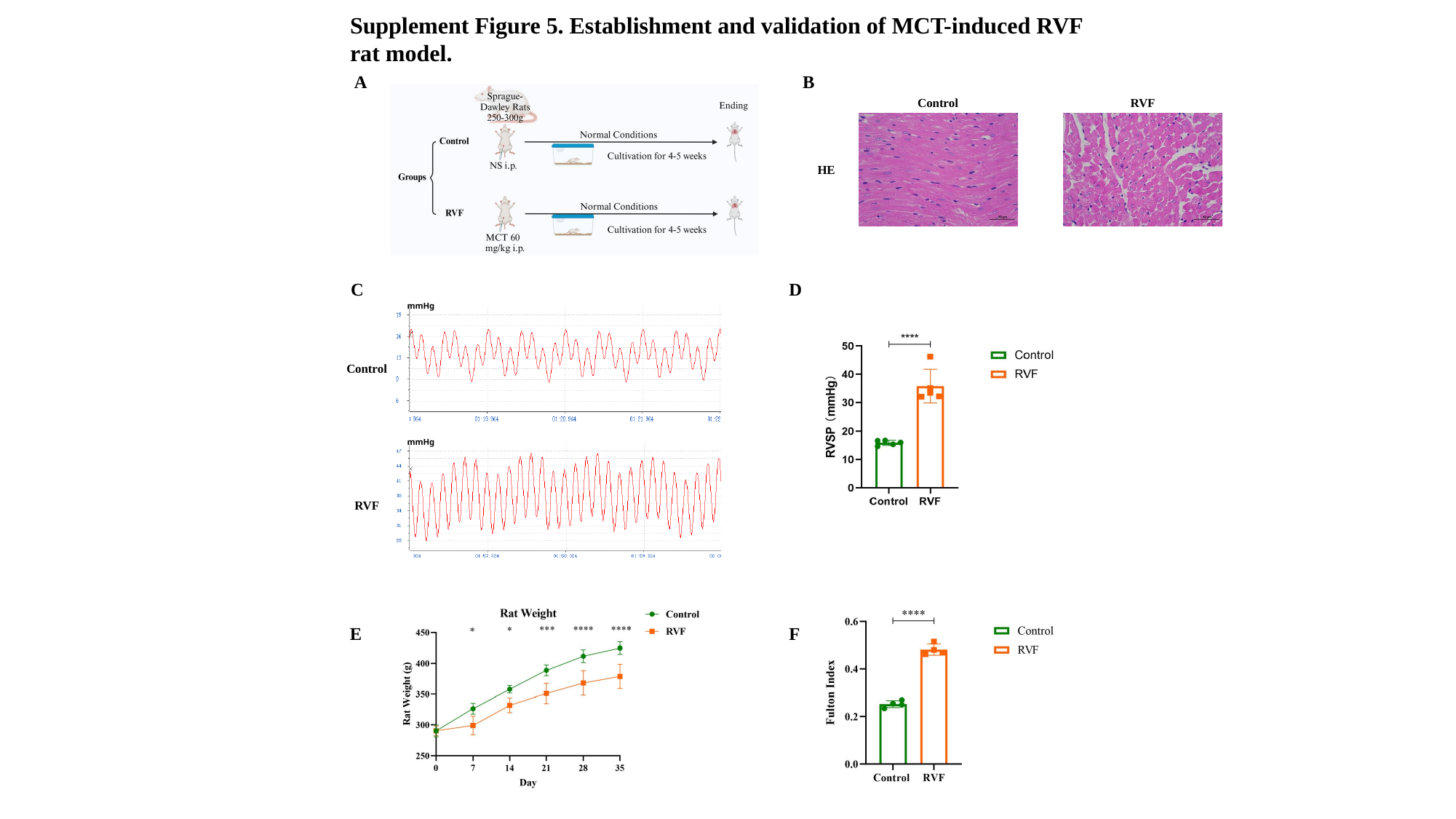

Supplement Figure 5. Establishment and validation of MCT-induced RVF rat model.
B
A
RVF
Control
HE
C
D
mmHg
Control
mmHg
RVF
E
F
