# Supplementary material for: Osteopontin regulates right ventricular failure through integrin ανβ3/PERK/CHOP-dependent inflammatory and apoptotic pathways
Source: Front Immunol. 2025 May 6;16:1569210. doi: 10.3389/fimmu.2025.1569210 (PMC12088963; doi:10.3389/fimmu.2025.1569210)
Supplement: Supplementary file 3 [file Presentation2.pptx]

## Slide 1
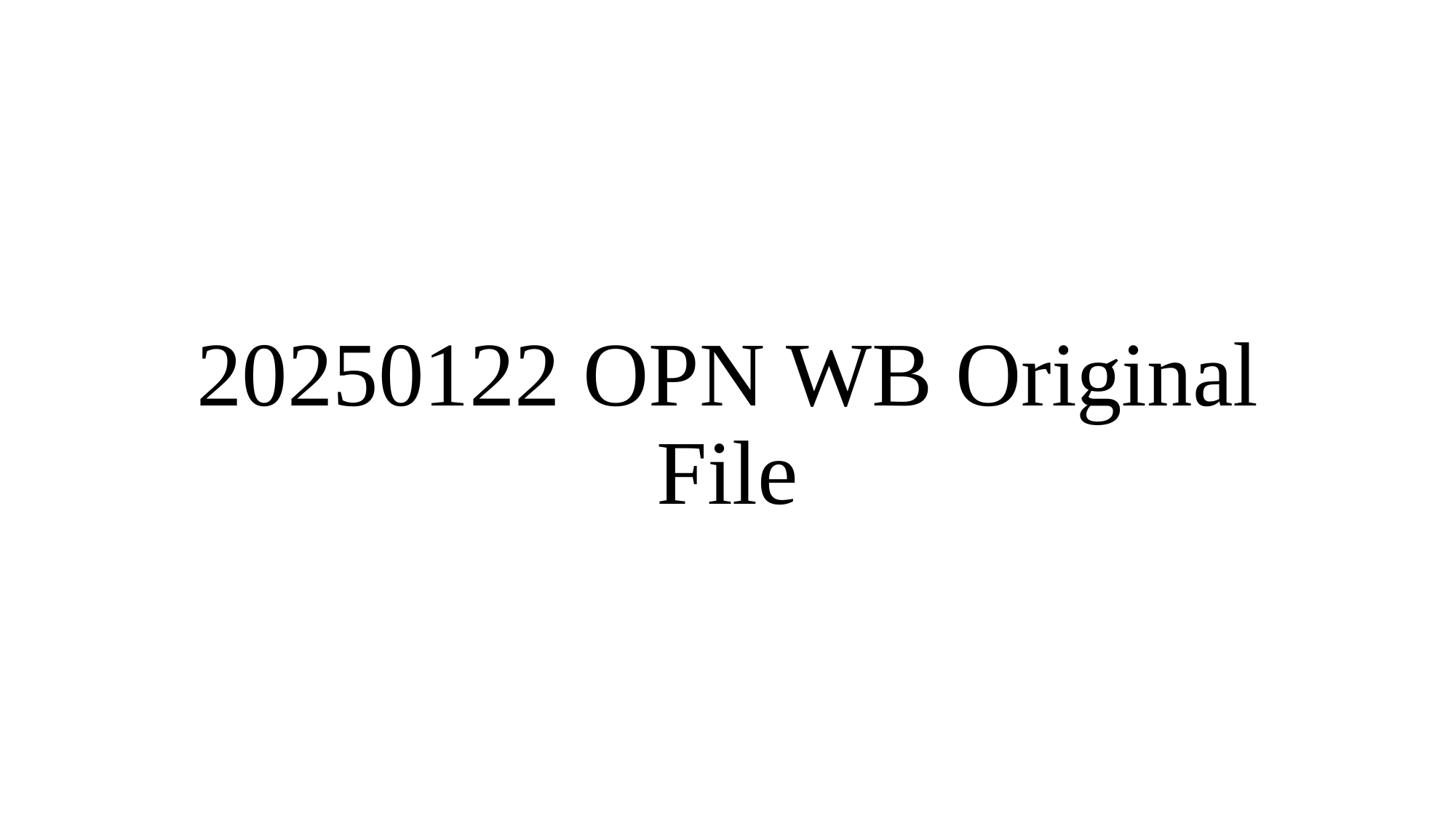

# 20250122 OPN WB Original File

## Slide 2
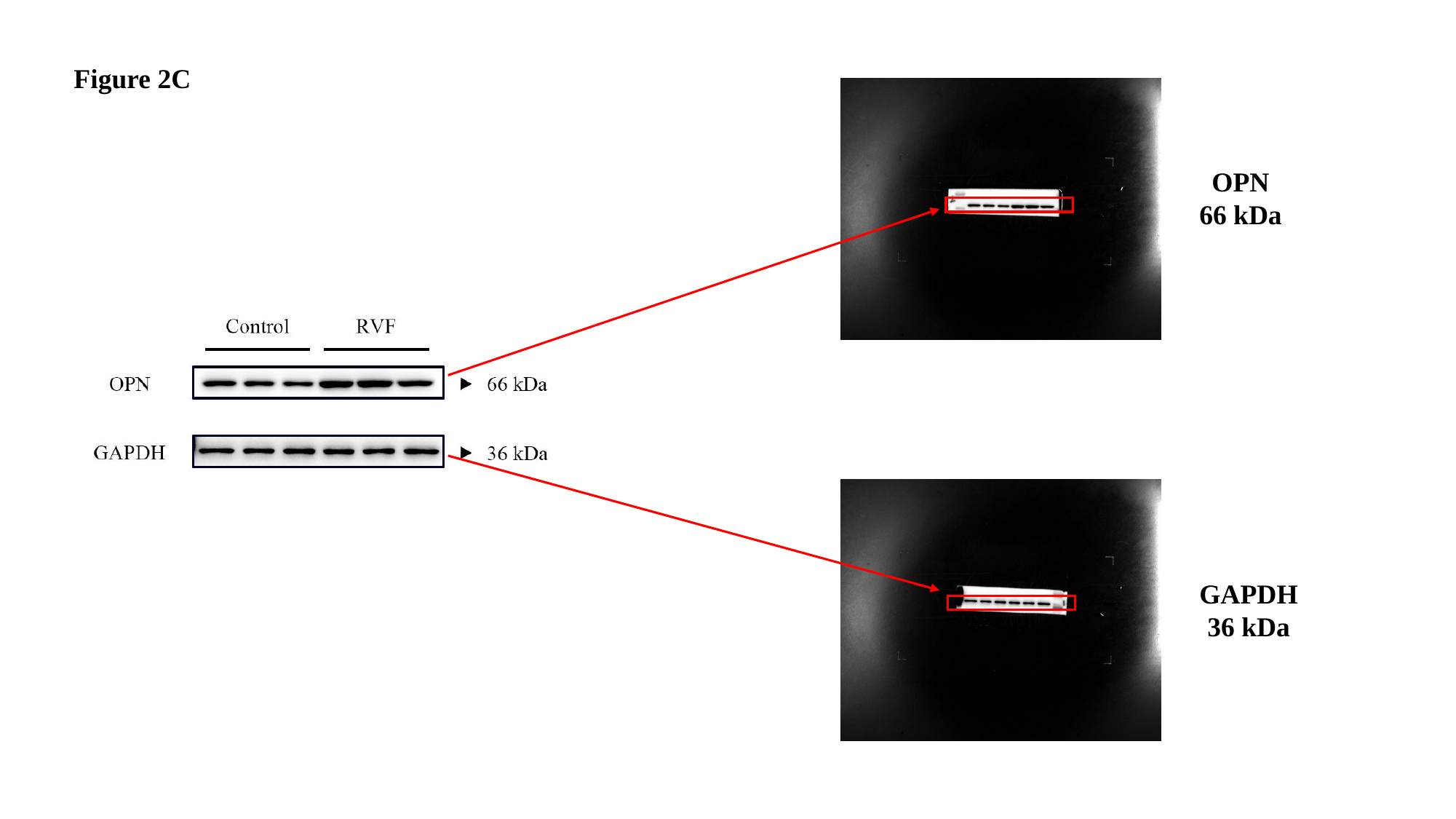

Figure 2C
OPN
66 kDa
GAPDH
36 kDa

## Slide 3
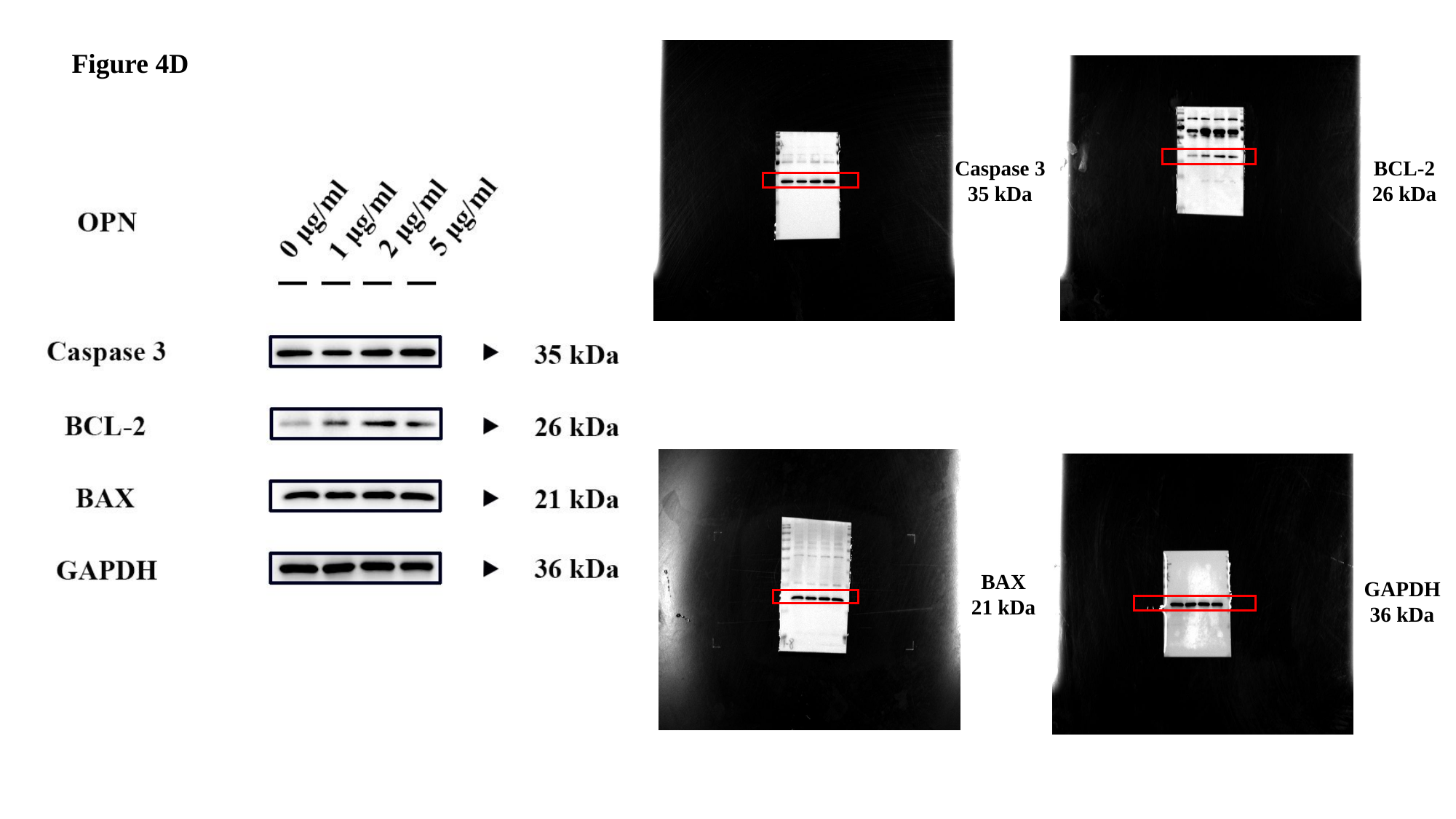

Figure 4D
Caspase 3
35 kDa
BCL-2
26 kDa
BAX
21 kDa
GAPDH
36 kDa

## Slide 4
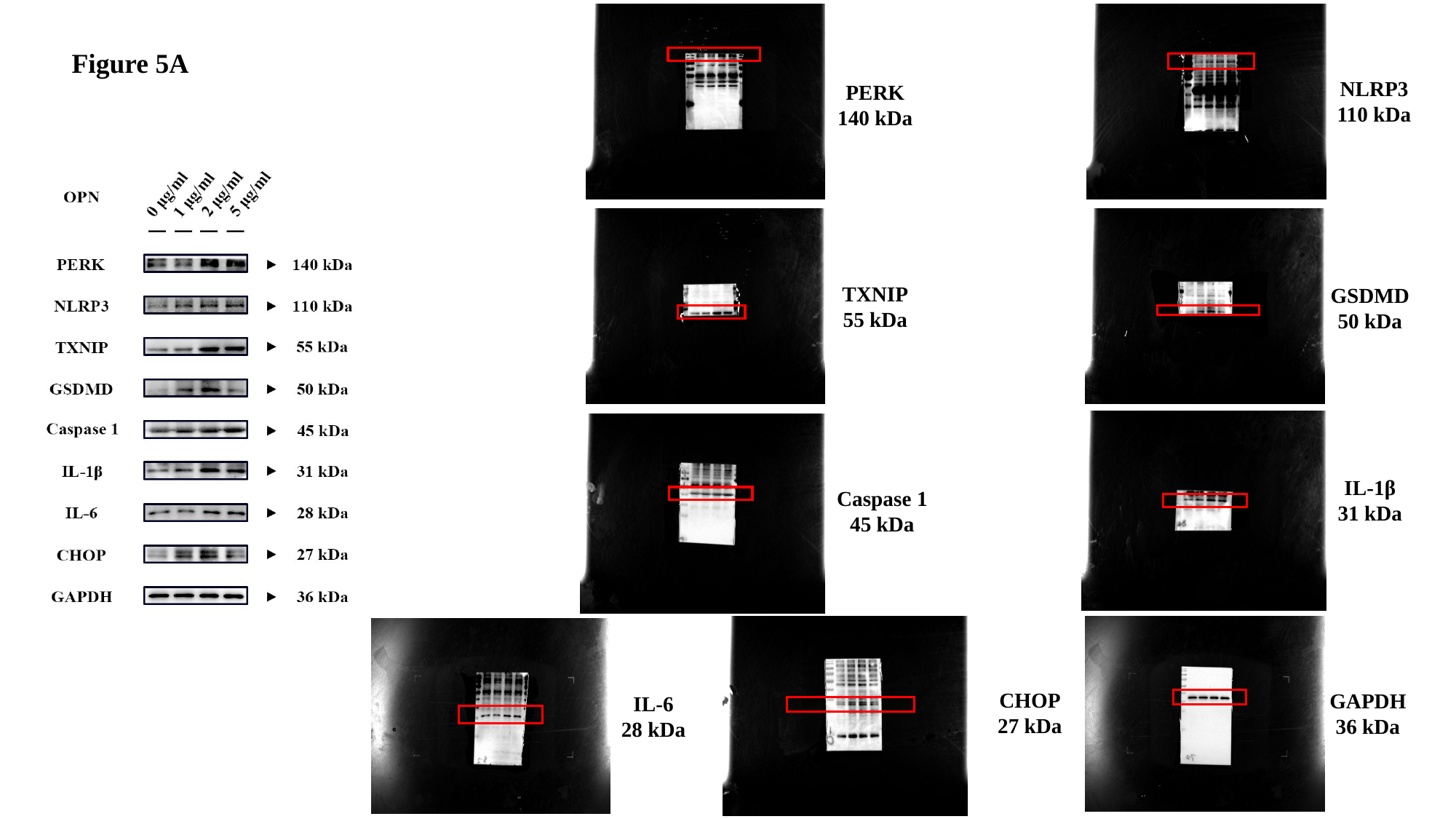

Figure 5A
NLRP3
110 kDa
PERK
140 kDa
TXNIP
55 kDa
GSDMD
50 kDa
IL-1β
31 kDa
Caspase 1
45 kDa
CHOP
27 kDa
GAPDH
36 kDa
IL-6
28 kDa

## Slide 5
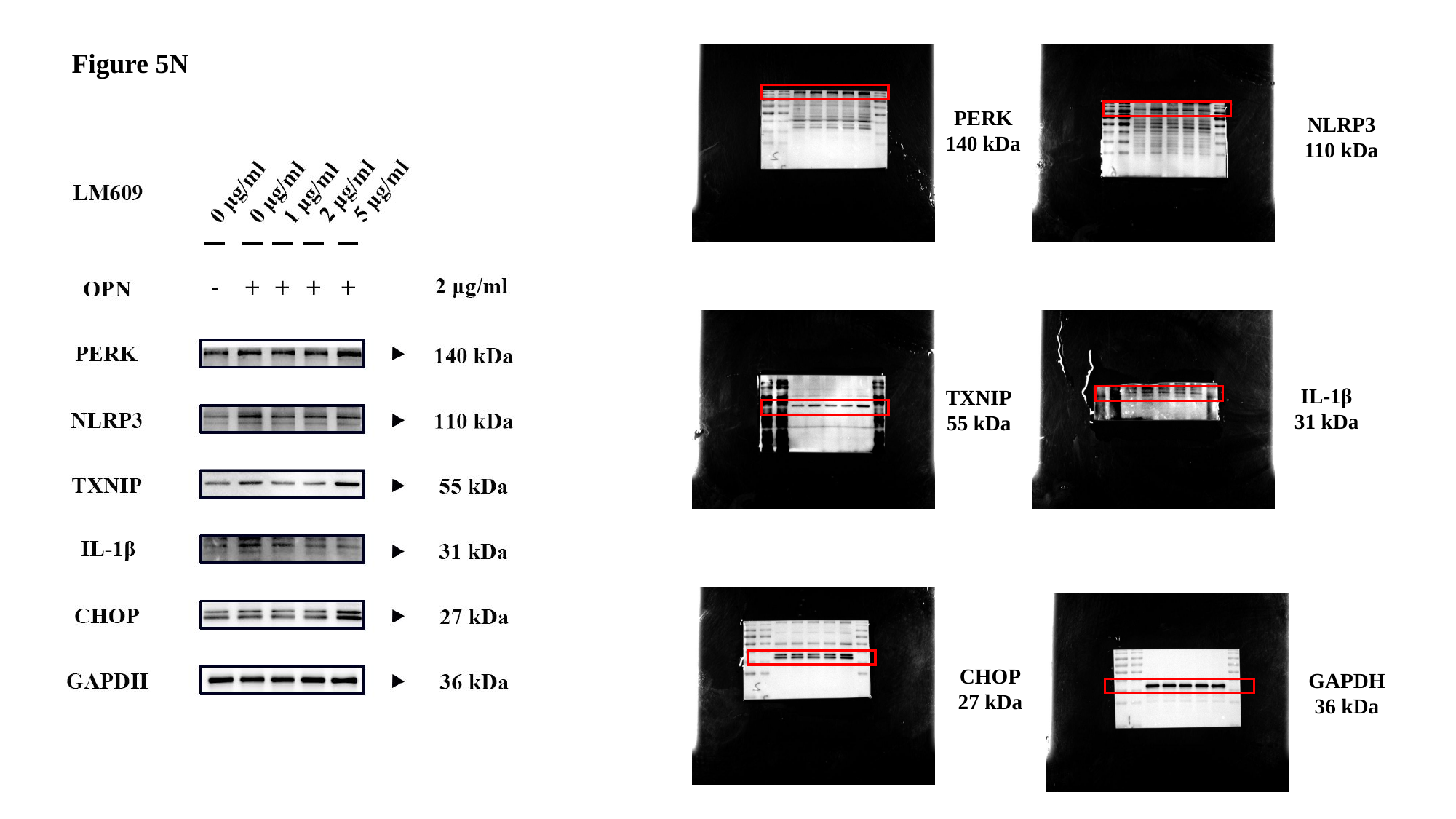

Figure 5N
PERK
140 kDa
NLRP3
110 kDa
IL-1β
31 kDa
TXNIP
55 kDa
CHOP
27 kDa
GAPDH
36 kDa
